# A global view of structure–function relationships in the tautomerase superfamily

**DOI:** 10.1074/jbc.M117.815340

**Published:** 2017-11-28

**Authors:** Rebecca Davidson, Bert-Jan Baas, Eyal Akiva, Gemma L. Holliday, Benjamin J. Polacco, Jake A. LeVieux, Collin R. Pullara, Yan Jessie Zhang, Christian P. Whitman, Patricia C. Babbitt

**Affiliations:** From the ‡ Department of Bioengineering and Therapeutic Sciences,; the ** Department of Pharmaceutical Chemistry, and; the ‡‡ Quantitative Biosciences Institute, University of California, San Francisco, California 94143 and; the § Division of Chemical Biology and Medicinal Chemistry, College of Pharmacy,; the ¶ Department of Molecular Biosciences, and; the ‖ Institute for Cellular and Molecular Biology, University of Texas, Austin, Texas 78712

**Keywords:** enzyme structure, evolution, protein evolution, protein sequence, protein structure, structure-function, enzyme superfamily, structure–function relationships, tautomerase superfamily

## Abstract

The tautomerase superfamily (TSF) consists of more than 11,000 nonredundant sequences present throughout the biosphere. Characterized members have attracted much attention because of the unusual and key catalytic role of an N-terminal proline. These few characterized members catalyze a diverse range of chemical reactions, but the full scale of their chemical capabilities and biological functions remains unknown. To gain new insight into TSF structure–function relationships, we performed a global analysis of similarities across the entire superfamily and computed a sequence similarity network to guide classification into distinct subgroups. Our results indicate that TSF members are found in all domains of life, with most being present in bacteria. The eukaryotic members of the *cis*-3-chloroacrylic acid dehalogenase subgroup are limited to fungal species, whereas the macrophage migration inhibitory factor subgroup has wide eukaryotic representation (including mammals). Unexpectedly, we found that 346 TSF sequences lack Pro-1, of which 85% are present in the malonate semialdehyde decarboxylase subgroup. The computed network also enabled the identification of similarity paths, namely sequences that link functionally diverse subgroups and exhibit transitional structural features that may help explain reaction divergence. A structure-guided comparison of these linker proteins identified conserved transitions between them, and kinetic analysis paralleled these observations. Phylogenetic reconstruction of the linker set was consistent with these findings. Our results also suggest that contemporary TSF members may have evolved from a short 4-oxalocrotonate tautomerase–like ancestor followed by gene duplication and fusion. Our new linker-guided strategy can be used to enrich the discovery of sequence/structure/function transitions in other enzyme superfamilies.

## Introduction

As the number of protein sequences from genomic and metagenomic sequencing continues to grow exponentially, the proportion of these sequences accessible to experimental function determination becomes vanishingly small, even when using high-throughput approaches. For enzymes, clues about reaction diversity that has evolved across the biosphere have contributed significantly to the development of principled and generalizable approaches to predicting the functional capabilities of proteins of unknown function (“unknowns”) and to using that information to identify informative targets for biochemical characterization or protein engineering. Mechanistically diverse enzyme superfamilies (1) (also referred to as functionally diverse superfamilies) represent about one-third of the universe of enzyme superfamilies (2). These superfamilies often contain more than 20,000 homologous sequences in which active-site machinery associated with a conserved aspect of catalysis is maintained through evolution, whereas specialized active-site and other structural variations evolve to enable many different reactions. Studying these systems offers powerful insight into the ways that nature has produced the enormously varied reactions necessary for life and provides a framework for inferring their functions. A small number of these superfamilies have now been studied on a large scale, revealing for each the structural and mechanistic foundation by which divergent evolution has produced many different reactions.

Here, we have described a global mapping of structure–function relationships among the members of the tautomerase superfamily (TSF)^4^ (3, 4). Although the TSF has not yet been examined on a large scale, its few known reactions reflect the presence of a treasure trove of enzymes with unusual properties. Of special interest, the unusual mechanistic use of an N-terminal proline offers a window into nature's use of “outlier” catalytic strategies (3, 5), broadening our still limited understanding of the chemistry supported by functionally diverse enzyme superfamilies. Especially important for the work described here is the relatively simple structural organization of TSF proteins, which has allowed us to uncover sequence variations among contemporary TSF sequences that may provide clues about how a simple ancestral scaffold may have diverged to produce widely varied reaction types and biological functions. TSF members are highly desirable experimental vehicles, as they do not require metal ions or coenzymes and are easily purified and expressed (4). As a result, computational predictions can be tested experimentally, enabling an iterative strategy for choosing and testing unknowns likely to inform a better understanding of structure, function, and mechanism across the superfamily.

**Figure 1.**
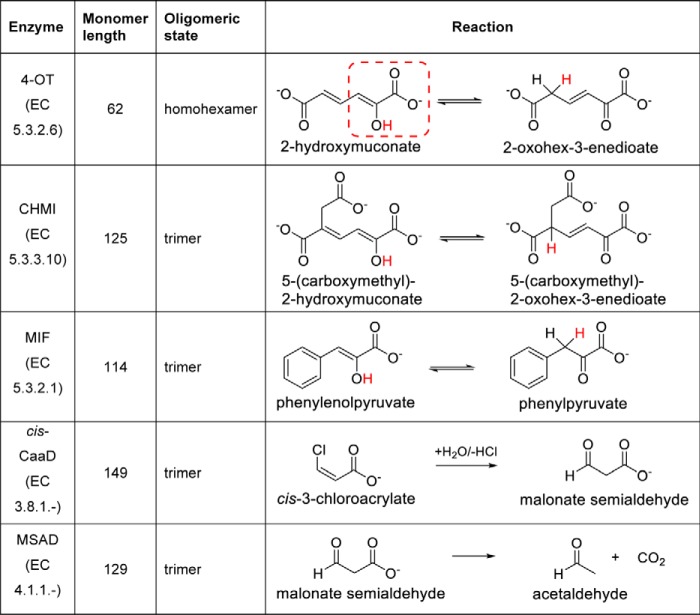
**Major types of reactions characterized in the TSF.** 4-OT (25), CHMI (8), PPT activity of MIF (11), *cis*-CaaD (27), and MSAD (28). The pyruvoyl moiety, which is the common functional group for the three tautomerase reactions of 4-OT, CHMI, and MIF, is *boxed* inside a *broken red line* for the 4-OT reaction. The proton that is transferred during each of these reactions is highlighted in *red*.

All biochemically characterized enzymes in the superfamily fall into five reaction types (Fig. 1), each of which uses the N-terminal proline either as a general base or a general acid (4, 6). All previous studies of characterized TSF members show a shared utilization of an N-terminal proline in their mechanisms, leading to an expectation that all TSF members would exhibit this feature. Notably, the common reaction catalyzed by three of these enzymes, 4-oxalocrotonate tautomerase (4-OT) (5, 7), 5-(carboxymethyl)-2-hydroxymuconate isomerase (CHMI) (8), and the phenylpyruvate tautomerase (PPT) activity of macrophage migration inhibitory factor (MIF) (9–11), is an enol–keto tautomerization of a pyruvoyl moiety in which Pro-1 has a low p*K_a_* value (∼6.4 in 4-OT) (7). The two other reaction types, catalyzed by *cis*- and *trans*-3-chloroacrylic acid dehalogenase (*cis*-CaaD and CaaD, respectively) (4, 12–14) and malonate semialdehyde decarboxylase (MSAD) (15), are more divergent mechanistically. Although these latter two enzyme-catalyzed reactions still utilize the N-terminal proline, it has a higher p*K_a_* value (∼9.2 in CaaD) (16) and functions as a general acid (15). In addition to its PPT activity, MIF is more commonly recognized as functioning as a pro-inflammatory cytokine in mammals (17–20). The PPT and the thiol-protein oxidoreductase activities (21, 22) of MIF are not involved in its cytokine activities (23, 24).

From a structural perspective, the functionally diverse members of the TSF share a small, structurally similar core domain that comprises a β-α-β motif. The smallest members of the TSF are composed of monomers ranging in length from 58 to 84 amino acids. For the handful of nonredundant structures available, oligomeric organization across the superfamily varies considerably and includes homo- or heterohexamers (a single β-α-β unit per monomer) (25, 26), trimers (two fused β-α-β units) (11, 27, 28), and a dimer (a single β-α-β unit) (29) (see Fig. S1 for details). Both the observed and as-yet uncharacterized variations among oligomeric organization may impact the *in vivo* catalytic capabilities of these proteins and contribute to the expansion of the functional repertoire of the TSF as well. Although most TSF members appear to be composed of either the core β-α-β motif or the fused duple form, the motif is also found appended to a limited number of non-ribosomal peptide synthetases such as indigoidine synthetase (30). For clarity of discussion, the short TSF members constructed of a single β-α-β structural motif are denoted in this work as a single β-α-β domain, and the longer TSF members consisting of two fused β-α-β structural motifs are denoted as two β-α-β subdomains.

Despite the attention that individual enzymes in the TSF have received, the full extent of the chemical and structural diversity across the superfamily remains unknown, as fewer than 30 members have been experimentally characterized. As with other functionally diverse superfamilies, this lack of experimental data prompts many questions. Do all TSF members have a catalytic Pro-1? Is the breadth of the superfamily reaction space confined to the five known reaction classes? Has nature used other arrangements for the core β-α-β motif in evolving the contemporary members of the TSF? How did the various subgroups evolve from what has been proposed to be the ancestral 4-OT–like enzyme (6, 22, 31, 32)? How did the multiple biological activities of MIF evolve? Is there a biological relevance for the reported enzymatic activities of MIF (23), such as the PPT activity (9–11)?

To begin to address some of these questions, we performed an all-by-all comparison of more than 11,000 nonredundant sequences of the TSF using sequence similarity networks to map key structure–function relationships among the superfamily members. The global views that resulted began to fill out the picture of structure–function relationships across the entire TSF, revealing new trends not previously available from small-scale studies. The results allowed us to classify the TSF into subgroups that exhibit different patterns of catalytic machinery and provide a map of its phylogenetic representation across the biosphere. We also addressed, in this work, how changes in reaction specificity–determining residues may have led to the evolution of varied functions. Phylogenetic reconstruction focusing on enzymes that show sequence similarity to two different subgroups (“linkers”), together with the other results reported here, supports the hypothesis that the *cis*-CaaD–like enzymes may have evolved from a 4-OT-like ancestor. Similarity networks from this paper, as well as other data, including multiple sequence alignments (MSAs) resulting from this study, are available for download from the Structure–Function Linkage Database (SFLD) (33) (http://sfld.rbvi.ucsf.edu/django/).^5^

## Results and discussion

### A large-scale comparison of 11,395 sequences reveals new structural and functional features of the TSF

**Figure 2.**
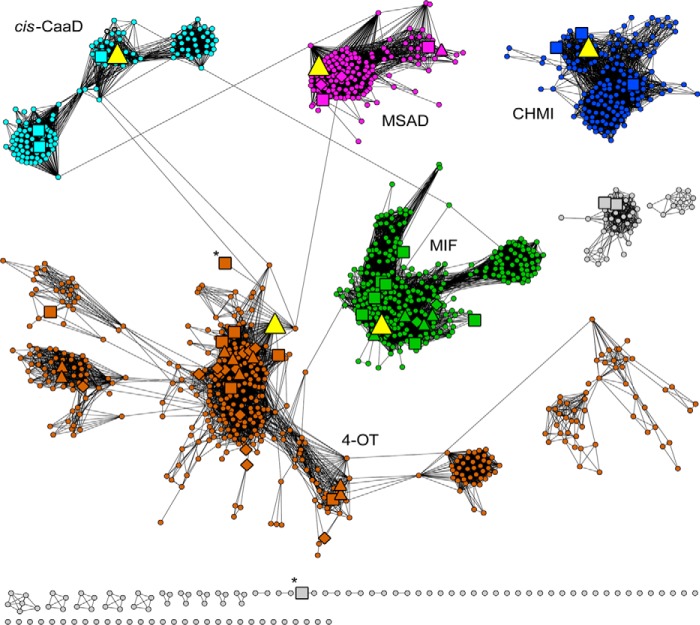
**Representative sequence similarity network of the TSF superfamily summarizes putative sequence–function relationships.** The 11,395 sequences of the TSF superfamily are represented by 1323 representative nodes, each binned into sets of TSF sequences at >50% pairwise identity. The threshold for drawing edges between representative nodes is 10^−11^ with geometric mean *E*-values used as scores (34). This threshold was chosen to optimize visualization of similarities within subgroups and the remote homologies between them (see “Experimental procedures”). The network was laid out using the Organic layout. For this layout, edge lengths, representing the degree of connectivity, qualitatively track with sequence dissimilarity. *Diamond-shaped nodes* have one or more experimentally characterized proteins with a SwissProt annotation (72); *square-shaped nodes* have one or more structurally characterized nodes; *triangular nodes* have one or more proteins that are functionally and structurally characterized. Nodes containing the sequence of a founder protein (described under “A large-scale comparison 11,395 sequences reveals new structural and functional features of the TSF”) are shown in *bright yellow*. (All of the founder enzymes within the *large yellow triangular nodes* have been biochemically and structurally characterized, although the experimental characterization of the *cis*-CaaD and MSAD founders are not designated as reviewed in SwissProt.) Although each subgroup is named for its founder reaction, the great majority of proteins in representative nodes in each subgroup have not been characterized; thus, an unknown proportion of each subgroup may not catalyze the founder reaction but instead may catalyze different reactions or have different or no physiological functions. The subgroups are named and colored as being most similar to their namesake founder proteins: 4-OT–like subgroup, *brown* (581 nodes representing 4472 nonredundant sequences); CHMI-like subgroup, *dark blue* (165 nodes representing 1767 nonredundant sequences); MIF-like subgroup, *green* (194 nodes representing 1655 nonredundant sequences); *cis*-CaaD–like subgroup, *cyan* (141 nodes representing 566 nonredundant sequences); MSAD-like subgroup, *magenta* (143 nodes representing 2050 nonredundant sequences). The *labels* next to each subgroup in the network denote the simple abbreviations for each founder reaction as given in Fig. 1. *Gray nodes* designate TSF sequences that have not been assigned to a named subgroup. Except for two larger clusters made up entirely of *gray nodes*, the majority of the *gray nodes* are in small clusters of ≤6 nodes or singleton nodes, indicating that they scored below the threshold required to connect them to the named subgroups. The two nodes marked with an *asterisk*, one in the 4-OT subgroup (*brown*) and one in an unassigned doubleton (*gray*), include chains (subunit α and subunit β, respectively) that comprise the heterohexamer CaaD (three α,β dimers form the active enzyme). However, as the α- and β-subunits share only 19% pairwise sequence identity, they are found in different nodes. We note that for many technical reasons (34) the visualized SSNs can only provide an estimate of sequence similarity and so should be considered as a starting point for developing hypotheses about sequence–function relationships rather than as definitive representations of such relationships. This issue is especially relevant in evaluating the significance of remote homologies among subgroups, discussed in detail under “Linkers between *cis*-CaaD and 4-OT subgroup identify a similarity path between them.”

All-by-all pairwise comparisons of 11,395 nonredundant sequences of the TSF were computed and used to generate a representative sequence similarity network (SSN) (34, 35) (Fig. 2). Each node in this network includes all TSF members that share >50% identity. Thus, the number of sequences comprising each node shown in the figure can be highly variable, ranging from 1 to 342 sequences/node. Using this network as an initial guide, the sequences were classified into Level 1 subgroups in which the sequences within each are more similar to each other than to the sequences in any other subgroup.

As indicated in Fig. 2, the majority of TSF sequences can be assigned to a Level 1 subgroup. Although the subgroups were defined based only on their sequence similarities, their mapping of the five known enzyme-catalyzed reactions shown in Fig. 1 to the network (Fig. 2) indicates that each belongs uniquely to a different subgroup. Based on this observation, the best-characterized protein from each subgroup was termed a “founder” protein (*highlighted* in Fig. 2). Each subgroup with a founder sequence was assigned a distinct color; other smaller sets of representative sequences and singleton sequences were not named or investigated further.

To provide a quantitative determination of the uniqueness of the subgroupings shown in Fig. 2, hidden Markov models (HMMs) (36) were computed for each named subgroup of the TSF and searched against all superfamily members to evaluate the degree to which each uniquely describes the subgroup from which it was computed (Figs. S2–S5). The results of this control experiment show that the HMMs generated for the Level 1 *cis*-CaaD, MSAD, CHMI, and MIF subgroups recovered the sequences of their associated subgroups at scores above the threshold used to uniquely define each subgroup, as noted in the figure legends of Figs. S2–S5, respectively, as expected, with only a few outliers in other subgroups.

As the diversity of the sequences of the 4-OT subgroup was too great to allow the generation of a high-confidence MSA for the entire subgroup, it was further subgrouped into four Level 2 subgroups (Fig. S6) for which alignments and HMMs could be reasonably generated. As with the main Level 1 subgroups, the Level 2 HMMs for the 4-OTs were mapped to their associated subgroups (Figs. S7–S10). The results showed that these Level 2 subgroup HMMs capture the majority of the subgroup members from which each was generated, although these HMMs have a larger proportion of cross-hits to other Level 2 subgroups of the 4-OTs than did the HMMs generated to the Level 1 subgroups. This is likely because of the greater diversity within the 4-OT subgroup relative to the diversity within each of the other Level 1 subgroups of the superfamily.

As reported for other functionally diverse enzyme superfamilies (37–40), only a small proportion of TSF members has been biochemically or structurally characterized, as indicated in Fig. 2. Note that only one functionally annotated sequence is required to depict a representative node as a large triangle (indicating an experimentally assigned function), even though that node may contain a large majority of uncharacterized sequences. This scarcity of functional information for most of the TSF subgroups represents a major barrier to understanding the range of contemporary reactions (or biological functions) that the TSF scaffold has evolved to support, limiting, in turn, knowledge that could be useful for the re-engineering of TSF members to catalyze new reactions in the laboratory (41).

#### 

##### Subgroupings may be useful for classifying newly discovered TSF members

Studies of experimentally characterized 4-OT Level 1 subgroup members have identified key active-site residues and patterns for some of the known reactions (5, 7, 8, 25, 42–45). These observations may be useful in predicting functions for uncharacterized TSF members that share these active-site patterns. Likewise, the assignment of newly discovered TSF members into the most appropriate subgroups can be achieved using the unique HMMs generated for each subgroup.

At the same time, many of these expected active-site patterns that are missing or differ among uncharacterized TSF sequences (data not shown) may suggest that they do not catalyze known reactions of the superfamily but may have other molecular functions instead. Future mapping of more detailed active-site variations or motifs to these networks may identify targets for experimental or structural characterization that could shed light upon as-yet undiscovered reactions of the TSF.

##### Domain structure and sequence length variation across the TSF

The domain structure of the TSF is relatively simple, with nearly all of the members represented by either a single β-α-β structural motif or as fusion proteins composed of two of these subdomains. Our results confirmed this pattern generally across the TSF; most sequences of the 4-OT subgroup are composed of a short monomer of 58–84 residues except for a small group of fused 4-OTs, discussed below. The great majority of the sequences assigned to the other four subgroups represent the fused form, which are roughly twice as long as the monomeric 4-OTs and are composed primarily of two fused β-α-β subdomains (Fig. S11**)**. For the longer fused superfamily members, the N- and C-terminal subdomains have diverged to produce unique sequence differences. The remaining superfamily sequences are of mixed size between the short and long proteins, largely due to variable sequence lengths in the regions that link the two β-α-β subdomains and at the C termini. As discussed in more detail below under “Linkers between *cis*-CaaD and 4-OT subgroup identify a similarity path between them,” the shorter 4-OT subgroup proteins align better with the C-terminal “half” of the longer fused proteins linking the *cis*-CaaD and 4-OT subgroups than they do with the N-terminal half. The domain structure of the fused proteins of the CHMI, MIF, *cis*-CaaD, and MSAD subgroups may indicate that they evolved by gene duplication and fusion from a simpler 4-OT-like ancestor.

### Some TSF sequences lack an N-terminal proline

**Figure 3.**
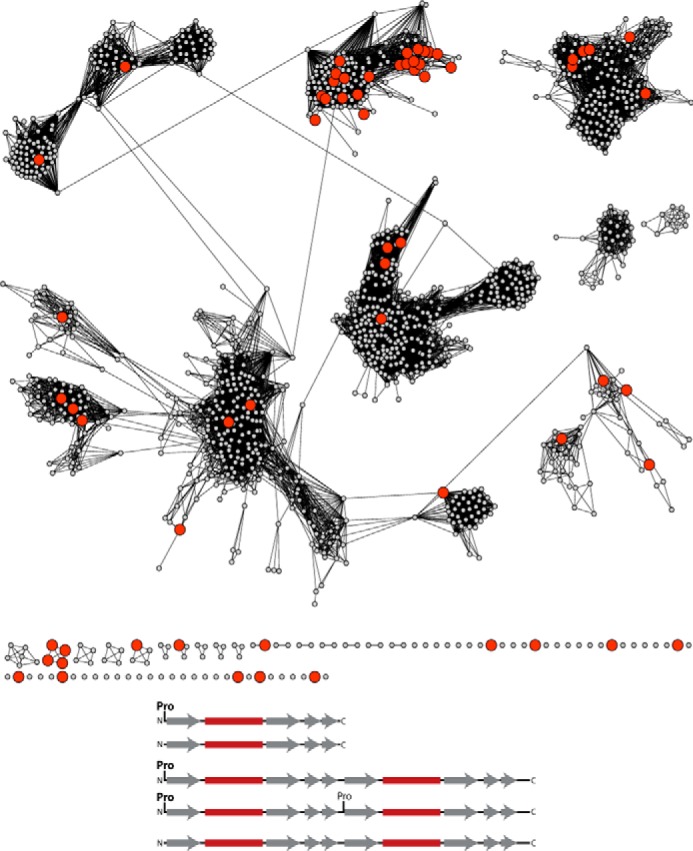
**Representative SSN showing sequences that lack an N-terminal proline.** *Top*, SSN as shown in Fig. 2, except that nodes containing one or more sequences that lack an N-terminal proline are colored *red. Bottom*, observed positions of the N-terminal proline (or its absence) in sequences of short and fused TSF members. *Gray arrows* and *maroon blocks* designate β-strands and α-helices, respectively.

Experimental work to date has suggested that the hallmark of the TSF, the catalytic N-terminal proline, is a mechanistic imperative. Surprisingly, our global analysis reveals that a significant number (346) of the TSF sequences lack Pro-1 (Fig. 3, *top*). (See “Experimental procedures” for a description of how this finding was verified.) Although missing this key Pro-1 residue, these sequences are clearly part of the TSF, as they align well with other superfamily members and retain conservation of other important active-site residues of the subgroup in which they are found. As none of these proteins have been characterized biochemically, their functions remain unknown. The majority of the non-Pro-1 superfamily members (294) map to the MSAD subgroup. Of the remaining 52 sequences, 13 map to the 4-OT subgroup, and the remaining sequences are scattered randomly across the entire network. Interestingly, the residues that are found in place of Pro-1 are not random variations. Serine, isoleucine, and alanine are the most abundant residues found that follow the initiating methionine (Fig. S12).

The absence of Pro-1 could reflect functional and/or mechanistic diversity in several ways. It is possible that an N-terminal amino group could function as a general base or acid. The experimentally characterized enzymes of the MSAD group, for example, which contains 85% of the non-Pro-1 proteins, have been shown to require a general acid in the enzyme-catalyzed reactions (15, 28, 46, 47). Or else, these proteins could perform catalysis via an as-yet uncharacterized catalytic mechanism that does not rely on the N-terminal group. Alternatively, some of these proteins may not function as enzymes, but rather may perform other biological functions, for example, they may act as regulatory proteins. A mechanistic interpretation of these observations is complicated by how the possible forms of non-Pro-1 TSF proteins might be utilized in the varied oligomeric forms currently known (or yet to be identified) in the superfamily (Fig. 3 and Fig. S1). An additional complicating issue is that some sequences will retain the initiating methionine and others will not (48). Clearly, experimental characterization of some of these non-Pro-1 sequences will be required to address these and other mechanistic questions.

The second proline in line 4 of the schematic shown in Fig. 3 (*bottom*) provides some support for the notion that the Fused 4-OT members may have evolved by gene duplication and fusion from a short 4-OT ancestor, as this proline is conserved in ∼60% of the fused members that are most similar to the founder 4-OT. There are 91 of these Fused 4-OTs, and based on an MSA of the 4-OT Level 2 subgroup 1 (data not shown), 57 of them likely have proline at the start of the second β-α-β subdomain.

### Phylogenetic representation in the TSF

**Figure 4.**
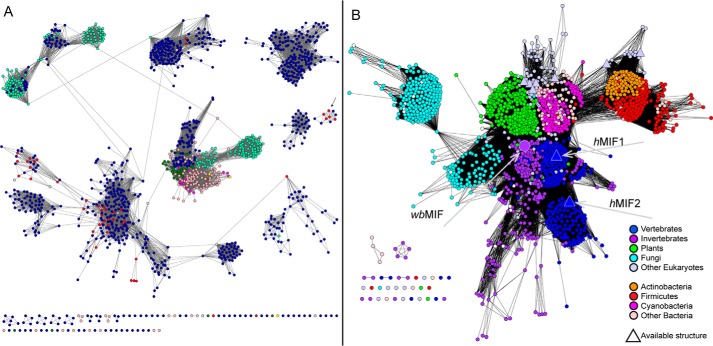
**Phylogenetic representation in the TSF.** *A*, sequence similarity network as shown in Fig. 2, except that the representative nodes are colored according to the dominant type of life: *red*, archaea; *dark blue*, bacteria; *green*, plants; *cyan*, fungi; *pink*, invertebrates; *yellow*, mammals; *magenta*, other vertebrates besides mammals. Also 26 *gray nodes* scattered among the *dark blue nodes* of the 4-OT subgroup come from environmental sequencing projects. The *arrow* marks an unnamed subgroup enriched in the archaeal sequences. *B*, a one-sequence-per-node similarity network of the MIF subgroup. Network nodes represent 1679 MIF sequences, 273 of which come from vertebrates. Coloring is according to type of life, as shown in the *key* except for the *white nodes*, which were not designated by type of life in the UniProt database. *Triangles* represent proteins with solved structures. The *large triangles* represent two characterized human proteins, MIF1 (UniProt P14174) and MIF2 (UniProt P30046), and the *large circle* represents a MIF from the human parasite *W. bancrofti* (UniProt O44786) as indicated by the *arrows*. The threshold for drawing edges between each node is 10*e*^−18^. The network shows two separate MIF groups in vertebrates; the sequences of the group containing the human MIF1 sequence are more similar to their invertebrate relatives than to the group containing the human MIF2 sequence.

As with many large superfamilies, the members of the TSF are found across the three domains of life. As shown in Fig. 4*A*, bacteria dominate in most of the TSF Level 1 subgroups, with a somewhat lesser relative representation in the *cis*-CaaD and MIF subgroups. Although a small set of 14 archaeal sequences was found in the MSAD subgroup, the 4-OT subgroup was significantly enriched in archaeal sequences (139 sequences, representing 71% of the 197 archaeal sequences in the TSF), suggesting an ancient origin for some of these 4-OT subgroup proteins. All of these sequences are uncharacterized except one, although its physiological function remains unknown (49). The small sequence cluster not named as a subgroup (marked by the *arrow* in the *far right* of Fig. 4*A*) was found to contain a substantial proportion of archaeal sequences as well. TSF members from eukaryotes are largely confined to the *cis*-CaaD subgroup, which is principally composed of proteins from fungi, and to the MIF subgroup. In the MIFs, the preponderance of proteins comes from eukaryotic phyla and includes substantial representation from fungi, plants, invertebrates, and vertebrates, including mammals. Although this subgroup includes significant representation from bacterial organisms, it does not appear to include sequences from the domain Archaea.

### Some MIF proteins in higher eukaryotes function as cytokines

Fig. 4*B* provides greater detail regarding how MIF sequences from varied organisms relate to each other. Among the MIFs, a total of 10 human MIF-like proteins are included in the network. Some of these may represent different splicing variants of MIF1 and of a second group of proteins known as MIF2 (previously known as d-dopachrome tautomerase (50)). Vertebrates represent 273 (16%) of the sequences in this SSN. Within the subgroup, a large set of proteins, including the human MIF1, are associated principally with cytokine activities (20) along with some enzymatic activities (*i.e.* PPT and protein-thiol oxidoreductase activities) (21–23). A second group of a similar size includes the human MIF2 (51). The human MIF1 and MIF2 structures are similar and their subunit topologies are almost identical, although there are differences in the active-site regions (19). Additionally, the functional properties of MIF2 overlap with those of MIF1, and the two may act cooperatively (52). MIF2 exhibits a tautomerase activity that requires Pro-1 (50) to use d-dopachrome, a compound with unknown biological relevance. Its relative tautomerase activity is about 10-fold less than that of MIF1 using the same compound. The MIF2-catalyzed tautomerization is followed by a decarboxylation reaction (23, 52). (The observation that tautomerization of l-dopachrome is involved in melanotic encapsulation, a hallmark of a primitive invertebrate defense pathway, suggests that the d-dopachrome activity might be a vestigial property (52).) Interestingly, our analysis of the network shown in Fig. 4*B* at more stringent *E*-value (a significance score describing how many matches are expected by chance when searching a database of a specific size) cutoffs for drawing edges (not shown) confirms the inference suggested in Fig. 4*B*, *i.e.* that the MIF1 proteins are more closely related to the invertebrate members of the subgroup than they are to the MIF2 proteins. Some of these invertebrate organisms are pathogenic (23).

A recent phylogenetic analysis of MIF homologs in plants revealed that an ancestral MIF-like sequence was present in the last common eukaryotic ancestor before plants diverged about 1.6 billion years ago (53) and noted that MIF-like proteins are found in many phyla, including bacterial ones. The large-scale view of MIF similarity relationships illustrated in the work described here and shown in Fig. 4*B* is consistent with those findings and indicates the relative proportion of MIF-like proteins found in bacteria, plants, and fungi. Many of these other organisms harboring MIF-like proteins do not have cytokines or the types of complex immune systems typically associated with cytokine activity and thus may have as-yet unknown functional roles. Another published phylogenetic reconstruction of experimentally studied MIFs noted that they may have evolved from an ancient defense molecule; in parasites MIF-like proteins appear to function as virulence factors (23). A MIF-like protein has been characterized biochemically from the human parasite *Wuchereria bancrofti* (wbMIF) and shows a low level of tautomerase activity (54). This latter analysis is the only known attempt to assign function to non-mammalian MIFs. Thus, both the molecular and biological functions and the importance of the broad representation of the MIFs across the biosphere remain poorly understood.

### Structure–function relationships between the 4-OT and cis-CaaD subgroups of the TSF

The results of our global study of the TSF show that almost all members of the functionally diverse TSF are united by the structure similarities of a common fold and conservation in key aspects of their chemistry and catalytic machinery (the exception being the 3% that lack an N-terminal proline). At the same time, the known TSF reactions are different from each other, raising the question how the fundamental structure–function relationship that defines the TSF has been modified by nature to evolve these different overall reactions.

Several issues made answering this question especially difficult. First, the extreme diversity of TSF subgroups relative to each other challenges our ability to make high-quality alignments between subgroups. For example, the sequences of the founder 4-OT (26) and *cis*-CaaD (32) enzymes (Fig. 1) share only 18% pairwise identity with each other. Second, the sparse experimental coverage of the superfamily severely limits our knowledge of the mechanistic and structural variations present in the TSF or even the breadth of reaction space it supports. Although these issues prevent a comprehensive study of TSF subgroup relationships, an initial examination of structure–function relationships between the best-studied members of the 4-OT and *cis*-CaaD subgroups was somewhat more tractable, as reported below.

#### 

##### Linkers between the 4-OT and cis-CaaD subgroups identify a similarity path between them

**Figure 5.**
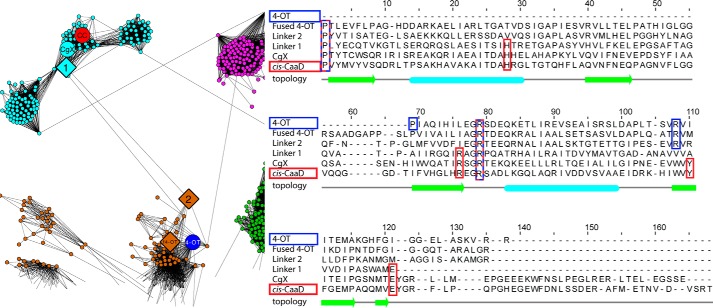
**Sequences in the 50% representative network that link the 4-OT and *cis*-CaaD subgroups.** *Left*, a more detailed version of the region in the 50% representative network (see Fig. 2) that links the 4-OT and *cis*-CaaD subgroups. Representative nodes are colored as described in the legend for Fig. 2 except that those containing founder 4-OT (labeled *4-OT*) and *cis*-CaaD (labeled *CC*) sequences are enlarged and colored *dark blue* and *red*, respectively. The nodes containing linker sequences Fused 4-OT (labeled *f4-OT*), Linker 2 (labeled *2*), Linker 1 (labeled *1*), and CgX are also enlarged. *Right*, structure-guided multiple sequence alignment of proteins labeled in the SSN enlargement on the *left*. Structures used and their PDB IDs: 4-OT, 1BJP; Fused 4-OT, 6BLM; Linker 2, 5UNQ; Linker 1, 5UIF; CgX, 3N4G; *cis*-CaaD, 2FLZ. The catalytic residues of the founder 4-OT, Pro-1, Arg-11, and Arg-39 (positions 69, 79, and 108, respectively, according to the numbering of the MSA) are *boxed* in *blue*, and catalytic residues of *cis*-CaaD, Pro-1, His-28, Arg-70, Arg-73, Tyr-103, and Glu-114 are *boxed* in *red* (positions 1, 28, 76, 79, 110, 121, respectively, according to the numbering of the MSA). Note that the short founder 4-OT aligns best with the second β-α-β domain of the five other proteins shown in this figure. The alignment shows that the active-site composition of the linker proteins becomes more *cis*-CaaD–like across the similarity path shown by the network. (Some catalytic residues in 4-OT and *cis*-CaaD come from different subunits, as described in the legend for Fig. 6.)

The SSN provided in Fig. 2 shows two representative edges that link the 4-OT and *cis-*CaaD subgroups, indicating a sequence similarity bridge between them. We identified representative nodes that formed the shortest path between the two founder sequences in each subgroup, highlighted in the *left panel* of Fig. 5. This path is anchored at either end by the representative nodes in which the founder 4-OT and *cis-*CaaD enzymes are located, with the linker nodes along the path proceeding from founder 4-OT to Fused 4-OT, to Linker 2, to Linker 1, to CgX, to founder *cis-*CaaD. (CgX is a structurally characterized *cis*-CaaD homolog with unknown function (31); it is an inefficient *cis*-CaaD, as it has much lower catalytic efficiency for this reaction than does *cis*-CaaD and it does not exhibit absolute specificity for the *cis*-isomer.)

As small numbers of edges linking highly diverse subgroups in functionally diverse superfamilies may be statistically suspect (34, 37), the links between the representative nodes shown in Fig. 5 were examined to address this issue further using more detailed SSNs. The results indicated that 48 statistically significant edges indeed link the 4-OT and *cis*-CaaD subgroups (see “Creation of linker control networks” under “Experimental procedures” for details).

A structure of a protein from each of these representative linker nodes was available, enabling the creation of a high-confidence structure-guided MSA that provided insight into the structural variations along this path. The *right panel* of Fig. 5 shows the structure-guided sequence alignment computed for these structures. The percent identity for each pair of sequences along the similarity path (Fig. 5, *right panel*) is 42% between 4-OT and the Fused 4-OT, 41% between Fused 4-OT and Linker 2, 26% between Linker 2 and Linker 1, 31% between Linker 1 and CgX, and 33% between CgX and *cis*-CaaD. These similarities are much higher than the 18% sequence identity obtained for a direct pairwise comparison between the founder 4-OT and *cis*-CaaD sequences in the alignment, which significantly enhances the quality of the alignment and the information it contains.

This MSA shows that the active-site composition of the linker proteins becomes more *cis*-CaaD–like in a stepwise manner across the similarity path from the founder 4-OT to *cis-*CaaD. Starting from the short 4-OT, the transition to the Fused 4-OT reflects the fusion of the single β-α-β unit represented by the short 4-OTs to form proteins with two β-α-β subdomains that are roughly twice as long as the short 4-OTs. These short sequences represent the majority of the proteins in the subgroup, whereas the Fused 4-OTs are more like the majority of the proteins in the other TSF subgroups, nearly all of which are composed of two fused β-α-β subdomains (Fig. S11). Among the characterized fusion proteins of the TSF, only two enzymes showed substantial 4-OT activity, Fused 4-OT and Linker 2. CHMI has been reported to have 4-OT activity as well (55) (Table 1). Fig. S6 indicates that the characterized Fused 4-OT protein matches best with 4-OT Level 2 subgroup 1.

**Table 1:**
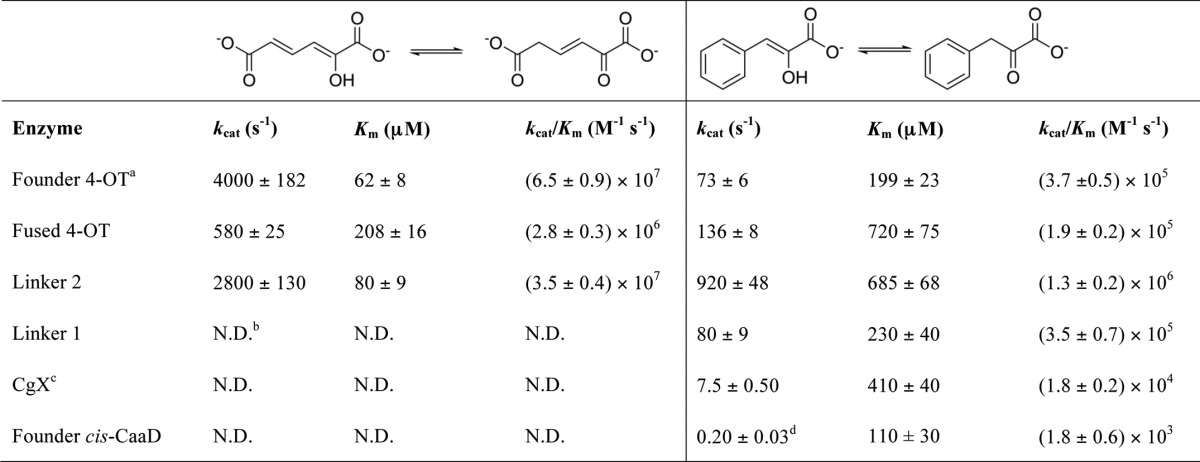
**Tautomerase activity across the linker proteins using 2-hydroxymuconate (left) and phenylenolpyruvate (right) as substrates**

*^a^* Ref. 26.

*^b^* Not detected.

*^c^* CgX and *cis*-CaaD show *cis*-CaaD activity. Linker 2 shows a trace of *cis*-CaaD activity. Linker 1 and Fused 4-OT do not show *cis*-CaaD activity.

*^d^* Ref. 56.

In the next step from Fused 4-OT, Linker 2 retains the 4-OT and Fused 4-OT–like sequence patterns, including conservation of Arg-39 (position 108 according to the numbering in Fig. 5 MSA), although it also exhibits some divergence away from them. Interestingly, as described under “Additional linkers among other TSF subgroups” below, both Linker 2 and the Fused 4-OT also show multiple structural similarities with other TSF subgroups. In contrast to Linker 2, Linker 1 shows significant *cis*-CaaD–like active-site properties, including a conserved His-28, Arg-70, and Glu-114 (positions 28, 76, and 121 according to Fig. 5 numbering) while losing conservation of 4-OT–like active-site residues, in particular Arg-39. Both CgX and *cis*-CaaD show completion of the remainder of the active-site machinery required for *cis*-CaaD activity, Tyr-103 and Glu-114 (positions 110 and 121 according to Fig. 5 numbering). Although they represent contemporary proteins, these linker sequences may provide hints about intermediate or “transitional” features resulting from divergence from ancestral genes. Based on the active-site variations observed in the linker set, along with the enriched representation of archaeal sequences in the 4-OT subgroup, we speculate that the Fused 4-OT subgroup members, the linker set, and founder *cis*-CaaD may have diverged from a short 4-OT–like ancestor.

##### Kinetic analysis showed loss of tautomerase activity in the enzymes linking 4-OT to cis-CaaD

As a first step in investigating the functional consequences of the linker transitions, we examined the enzymes in the structure-guided MSA (Fig. 5) for tautomerase activity using 2-hydroxymuconate (2-HM) and phenylenolpyruvate (Table 1). These substrates were selected because both are processed by 4-OT, and phenylenolpyruvate is processed by *cis*-CaaD. (The tautomerase activity of *cis*-CaaD has long been regarded as an evolutionary vestige from a tautomerase-like ancestor (56).)

**Figure 6.**
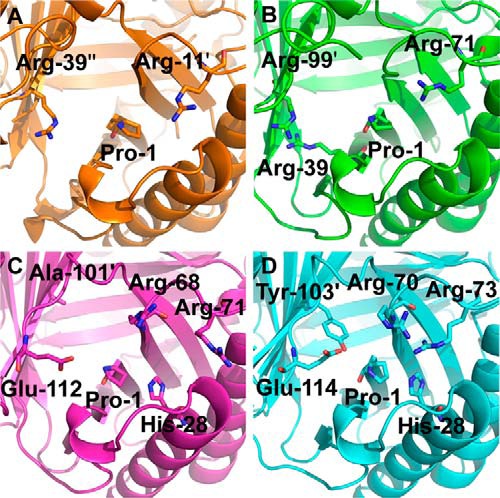
**Structural comparison of conserved active-site residues in Linker 1 and Linker 2 with respect to the known catalytic residues of founder 4-OT and *cis*-CaaD.** The structures for each of these proteins are the same as those included in the structure-guided MSA shown in Fig. 5. The unprimed, primed, and doubly primed residues indicate that they come from different subunits. *A*, founder 4-OT. Pro-1 is positioned between Arg-11′ and Arg-39". This arrangement allows binding of the dicarboxylate substrate 2-HM by both arginine residues and proton transfer by Pro-1 from the 2-hydroxyl group of 2-HM to C5. *B*, Linker 2. The active-site architecture of Linker 2 is much like that of founder 4-OT. Arg-71 and Arg-99′ (*boxed* in the MSA) are structurally equivalent to Arg-11′ and Arg-39" in founder 4-OT, respectively. Both arginine residues are present in the second β-α-β subdomain of the Linker 2 monomer, which explains their very different position in the protein sequence. Linker 2 also has an Arg-39 in its first β-α-β subdomain, which structurally forms part of the wall of the active site near Arg-99′. Its proximity to both Arg-99′ and Pro-1 could signify a potential role in catalysis. *C*, Linker 1. Linker 1 exhibits an active-site architecture very different from that of the founder 4-OT and Linker 2 and instead is more similar to the active site of *cis*-CaaD (in *D*). One important difference is the absence of an arginine residue that is structurally equivalent to Arg-39" in founder 4-OT and Linker 2. Instead, this arginine residue (Arg-68) appears to be repositioned much closer to Arg-71 (structurally equivalent to Arg-11′ and Arg-71 in founder 4-OT and Linker 2, respectively). The other residues that are highlighted, His-28, Ala-101′, and Glu-112, are structurally equivalent to His-28, Tyr-103′, and Glu-114 in founder *cis*-CaaD. Except for the missing Tyr-103′, which has Ala-101′ in that position, the catalytic machinery of founder *cis*-CaaD is complete in Linker 1. *D*, founder *cis*-CaaD. The active site of *cis*-CaaD, showing its known catalytic machinery, is composed of Pro-1, His-28, Arg-70, Arg-73, Tyr-103′, and Glu-114.

An examination of changes in active-site architecture in the linker proteins (Figs. 5 and 6) suggested to us that Linker 1, *cis*-CaaD, and CgX would not process a dicarboxylate substrate such as 2-HM. As a result, the monocarboxylate substrate phenylenolpyruvate would be expected to be more informative for gauging changes in the tautomerase activity of the linker proteins. Indeed, both Fused 4-OT and Linker 2 were shown to be proficient tautomerases with 2-HM, whereas Linker 1, *cis*-CaaD, and CgX showed no activity. Compared with 4-OT, Linker 2 exhibited only a 2-fold drop in *k*_cat_/*K_m_*, whereas Fused 4-OT showed a 23-fold drop in *k*_cat_/*K_m_*. Nonetheless, both are highly efficient tautomerases using 2-HM. In contrast, tautomerase activity with phenylenolpyruvate was detected for all of the enzymes. There was a stepwise loss of this activity going from 4-OT and the 4-OT–like enzymes (Fused 4-OT and Linker 2) to Linker 1, CgX, and ultimately *cis*-CaaD. Especially noteworthy, the variations in specific functionally important residues correlated with this activity trajectory (Fig. 6). Taking together the active-site transitions illustrated in Fig. 5 along with the kinetic data given in Table 1, these linker sequences may provide hints about ancestral features relevant to the evolution of the *cis*-CaaD and 4-OT–like proteins.

### Phylogenetic reconstruction of the linker set

**Figure 7.**
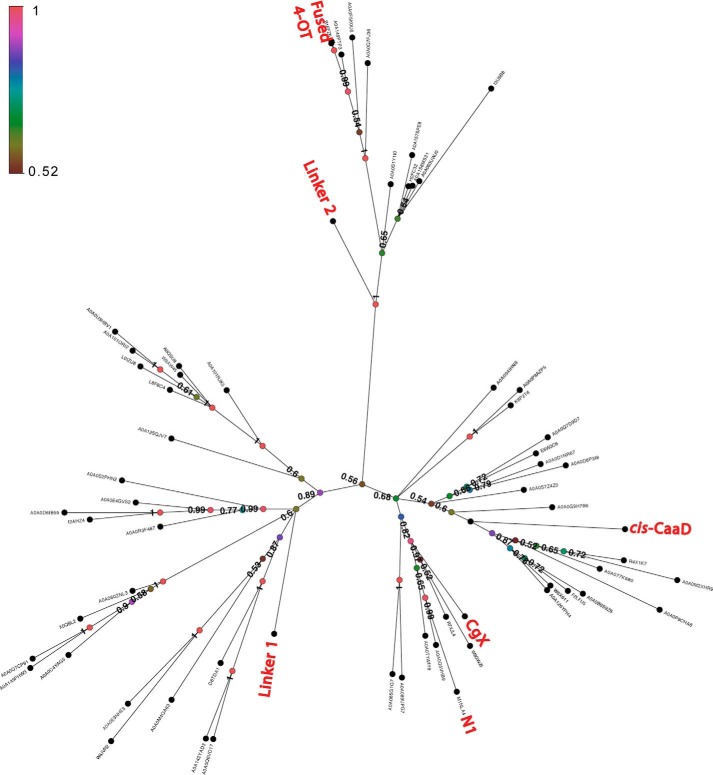
**Phylogenetic tree of the 4-OT and *cis*-CaaD linkers from the 50% representative SSN.** The tree was calculated by a Bayesian analysis of 63 sequences from an expanded linker set of proteins composed of two β-α-β domains. Posterior probabilities, designated according to the *color key*, are indicated for all interior nodes. The main branches associated with sequences from representative nodes of linkers are labeled. As the single-domain short 4-OTs contain only about half of the sequence information of the two subdomain fused proteins of the linker set, they were not included in the tree. Of the two additional linkers, linker N1 and linker N2 (described under “Identification of linkers” in the “Experimental procedures”), linker N1 is labeled in the tree. Linker N2 does not appear in the tree because it is a short 4-OT.

To examine the relationships across the linker set using an independent approach, we generated a phylogenetic tree. Guided by SSNs created to expand the linker set to include specific proteins from the representative nodes shown in Fig. 5, as well as their closest homologs, we identified 63 sequences for inclusion in the tree (see “Experimental procedures”). The MSA composed of these sequences was used to compute this tree and is provided in Fig. S14. The unrooted phylogenetic tree of these proteins is provided in Fig. 7. (Short 4-OT sequences were not included in the tree because these sequences contain only about half as much information as the rest of the fused sequences of the linker set, raising complications in interpreting the tree.)

The probability values at the leaf branches of the tree are generally of high quality within most of the major clades, although the diversity of the sequences is too great to generate an MSA of sufficiently high confidence to support rooting the tree. However, even the poorer significance values at the interior nodes provide support for the relationships along the similarity path given in Fig. 5. Those data, along with the tree and the kinetic results (Table 1), are generally consistent with each other, providing added confidence in our approach for dissecting the details of the structure–function relationships between the 4-OT and *cis*-CaaD subgroup enzymes. Without the global context provided by a TSF-wide SSN, identification and analysis of the linker set would have been difficult to achieve.

As noted in earlier sections, two lines of evidence provide qualitative support for the hypothesis that the contemporary TSF evolved from a short 4-OT–like ancestor. These include the likely (by parsimony) emergence of Fused 4-OTs from a simpler ancestral type composed of a single β-α-β subdomain and the enrichment of archaeal sequences in the short 4-OT subgroup relative to the lack of archaeal members in the *cis*-CaaD subgroup. We cannot, however, assume a unidirectional evolution from 4-OT to *cis-*CaaD from that data alone. Even though the phylogenetic tree provides new insight into the relationships between these two subgroups, the tree cannot be used to resolve the direction of their evolution, both because the tree is unrooted and because the short 4-OT proteins are not included in the tree.

Interestingly, the phylogenetic tree suggests additional groups of sequences that might be added to the linker set originally identified in this work. In the absence of available structures for proteins in these diverse unlabeled clades of the tree, the identification of active-site variations that might offer new clues about the functions of these proteins remains beyond the scope of this work. The positioning of these sequences in the tree, however, suggests that some of them may be good targets for structural characterization that might help to fill out the similarity path in more detail. As a case in point, the identification of Linker 1 led to our targeting of that protein for structural characterization (PDB ID: 5UIF) (57), allowing us to include it in the structure-guided alignment shown in Fig. 5.

### Additional linkers among other TSF subgroups

Further studies to gain insight into structure–function relationships across the TSF would undoubtedly benefit from employing a broader context that includes all of the subgroups. For the work reported here, the extreme sequence diversity among the five TSF subgroups along with the scarcity of structures across the superfamily prevented the creation of a high-confidence MSA sufficient to support phylogenetic reconstruction of the entire superfamily. Characterization of additional structures that better sample all TSF subgroups would aid in generating a TSF-wide MSA. In the future, the addition of more TSF sequences from metagenomic data may also contribute to the creation of a statistically significant superfamily-wide tree.

**Figure 8.**
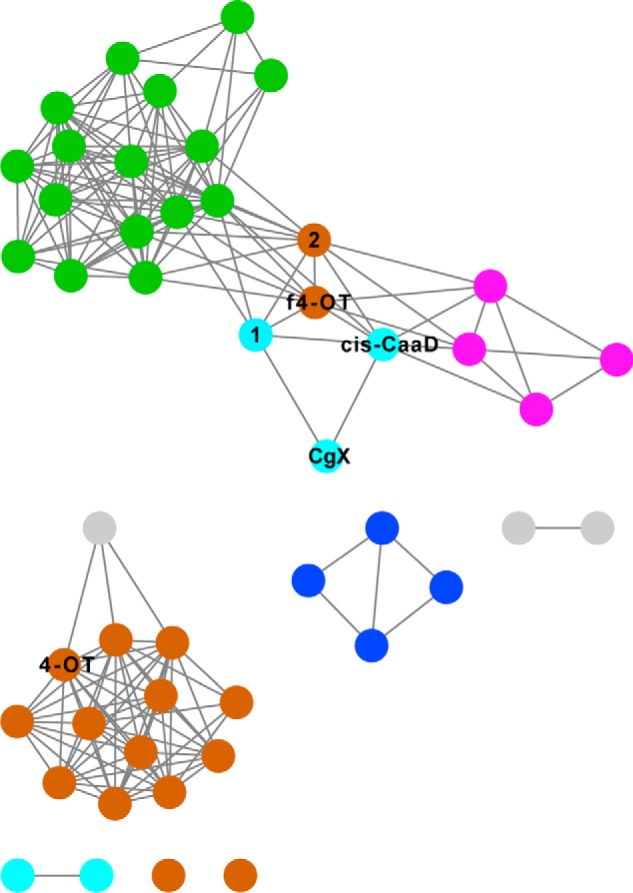
**Structure similarity network of the TSF.** Each node represents a single structure, colored by subgroup as described in the legend for Fig. 2. The threshold for drawing edges between structures is a TM-align score of 0.8. (A TM-align score of 0.5 is suggested to be statistically significant (66).) This network contains 48 structures for which the PDB accession codes are listed in File S1. The *labeled nodes* refer to proteins identified in the 4-OT/*cis*-CaaD linker set shown in Fig. 5.

In addition to linkers between the 4-OT and *cis-*CaaD subgroups, the representative SSN shown in Fig. 2 hints at additional putative links among other TSF subgroups, although the significance of these few linking edges is uncertain without further analysis such as reported here for the 4-OT and *cis-*CaaD linker set. As a next step toward expanding this larger context beyond the 4-OT and *cis*-CaaD subgroups, we used the currently available structures to compute a structure similarity network across the TSF as structure similarity can be identified at greater levels of sequence diversity than can be achieved from sequence comparisons (58). Based on this network, an initial, albeit cursory, view of those subgroup relationships is shown in Fig. 8.

This network reveals connections between subgroups that are not readily apparent from the TSF SSN (Fig. 2). Especially interesting is that two of the key structural connections linking the 4-OT and *cis-*CaaD subgroups, Fused 4-OT and Linker 2, also exhibit multiple links to the MIF and MSAD subgroups as well as links between the *cis*-CaaD subgroup and the MIF and MSAD subgroups. Thus, the structure similarity network extends the context in which we can hypothesize linkers among most of the subgroups, with the CHMI subgroup currently representing an outlier subgroup (also found in the SSN (Fig. 2)). We speculate that the central position of Fused 4-OT and Linker 2 in the structure similarity network offers a starting point for new studies aimed at addressing the evolution of the contemporary TSF subgroups from an ancient scaffold.

The 4-OT subgroup members principally composed of short single β-α-β domain proteins do not connect with the Fused 4-OT structure node or with Linker 2 in Fig. 8. As with the phylogenetic tree, the lower information content of the short sequences of the 4-OT subgroup complicates the interpretation of their relationships to other linkers and subgroups in the structure similarity network and likely results in its separation in the network layout shown in Fig. 8. Thus, we cannot easily interpret the relationship between the short 4-OTs and the Fused 4-OTs with other linker sequences from either the sequence or structural similarity network. This issue is also reflected in and addressed in different ways in other analyses presented in this work.

### Summary

The results of this analysis showed for the first time, a global view of structure–function relationships across the TSF. As with other superfamilies that have been computationally characterized in recent years, a large majority of the TSF members have not been experimentally characterized in any way, so that generating high-confidence functional hypotheses for unknowns based on their similarity to the few available “knowns” remains difficult and likely to be plagued by high levels of misannotation (59). Surprisingly, our global analysis of known TSF sequences revealed that about 3% of the superfamily sequences lacked an N-terminal proline, raising important questions about the long-held assumption that a Pro-1 residue is required for catalytic activity. Guided by structure-based alignments, we identified linker sequences that best connect active-site variations between the 4-OT and *cis*-CaaD subgroups, providing an indication of structural “transitions” that may distinguish the different reaction classes in each. The type of linker analysis described in this work offers a more detailed, information-rich strategy for comparing the variations between contemporary homologous proteins, thus contributing to biological and mechanistic insights in ways that are not so accessible from simple pairwise comparisons. Phylogenetic reconstruction of these linker relationships is consistent with the network-based linker analyses. Finally, the structure-based identification of links among most of the TSF subgroups defined in this work provides new directions for examining how each subgroup may have evolved in different ways to produce new catalytic and biological functions.

## Experimental procedures

### Sequence data collection

The TSF has sequence entries annotated as tautomerases in many of the major protein databases. InterPro (60) identifies the TSF by the following signatures: IPR028116, IPR001398, IPR015017, IPR004220, and IPR014347. These sequences were added to the SFLD (33) on August 16, 2016. Removal of redundant sequences produced a final, nonredundant set of 11,395 sequences for further analyses.

### Construction of sequence similarity networks

Sequence similarity networks were constructed for the TSF using algorithms taken from Pythoscape software (35) and tailored for use with the SFLD and its hardware environment. Sequences to be considered for each network were compared pairwise in an all-by-all manner using the BLAST algorithm (61) with an edge recorded if BLAST reported an alignment with an *E*-value more significant than 10^−5^. Edges were then pruned by thresholding the *E*-value scores at which the edges were drawn. Two types of SSNs were computed: a one-sequence-per-node network (also called a 100% identity network) and representative SSNs. Fig. 4*B* is the only 100% identity network presented in this work. Representative networks were built using a similar protocol, except that each node represents one or more TSF sequences identified by the CD-Hit algorithm (62) at a pairwise percent identity cutoff reported in the respective figure legends of each representative network. This reduction of many similar nodes into single nodes allowed the creation of representative networks that could be analyzed at several levels of detail based on the percent identity cutoff chosen for binning sequences in each representative node. The *E*-value thresholds used for drawing edges between representative nodes was determined from the average of the *E*-value scores between the sequences contained in each pair of nodes. For this study, representative networks were generated for the TSF using percent identity cutoffs at ≥50% (in Figs. 2, 3, 4*A*, and 5 and Figs. S2–S5); for smaller subgroups or subsets of TSF sequences, representative SSNs were computed using ≥90% pairwise identity cutoffs (in Figs. S6–S10 and S13). For each percent identity cutoff, the number of sequences in the representative nodes can be highly variable, as reported in the text. A representative edge between each pair of representative nodes was recorded if the one-sequence-per-node network recorded any edges between sequences in one representative node with sequences in the other. The score for each representative edge was computed as the geometric mean of the BLAST *E*-value scores of all recorded one-sequence-per-node edges between the connected representative nodes.

Networks were analyzed using a thresholded approach such that edges were drawn between each pair of nodes if the similarity between those sequences was better than a statistical significance threshold chosen to illustrate the similarity relationships in the SSN at different levels of detail. The edge metric used for these comparisons was the BLAST *E*-value, used as a score (34). This thresholded approach allowed the examination of SSNs across a range of *E*-values. For the network(s) shown in the 50% representative network depicted in Fig. 2 (and also in Figs. 3, 4*A*, and 5), the set of thresholds (*E*-value scores) was manually examined between 10^−2^ and 10^−20^ to identify a threshold estimated to be near optimal for displaying the *n*-dimensional relationships among subgroups in a compressed form as two-dimensional images. All networks shown in this work were visualized in Cytoscape (63) using the Organic layout. The length of the edges using this layout correlates with the order of connectivity and generally tracks with dissimilarity (34). Note that the representative nodes comprising the MIF subgroup in the SSN figures presented in Figs. 2, 3, 4*A*, and 5 were moved slightly because they were overlaid on an edge connecting the MSAD and 4-OT subgroups, giving the erroneous inference that that edge also connected to the MIF subgroup. As edges visualized with the Organic layout do not directly denote similarity distance but instead correlate with the number of pairwise connections between nodes, this manipulation did not alter the quantitative representation of the data.

### Division of the TSF into subgroups based on sequence similarity

An optimal threshold of 10^−11^ was chosen for subgrouping the sequences of the TSF into Level 1 subgroups in which the similarities in each were greater within each subgroup than between subgroups. The degree to which each subgroup is distinct from other subgroups (at the *E*-value threshold at which it was visualized) was verified using MSAs and HMMs (36) as described in the text accompanying Figs. S2–S10. Sequence clusters that were too small or too diverse for generation of a useful MSA and HMM were not further named or classified.

As the diversity of the Level 1 4-OT subgroup was too great to allow the creation of a single high-quality MSA depicting the entire subgroup, these sequences were further subgrouped, guided by a new and more detailed representative network generated from the Level 1 4-OT subgroup. Each representative node was composed of the 4-OT sequences binned at ≥90% pairwise identity. This network is visualized at an *E*-value threshold of 10^−18^ (shown in Fig. S6). Using a procedure similar to that described for the generation of the Level 1 subgroups, four of the largest clusters were defined as Level 2 subgroups (denoted in the SFLD as groups 1–4 and listed below the Level 1 4-OT subgroup in the SFLD). MSAs and HMMs for these Level 2 subgroups verified their uniqueness (Figs. S7–S10). using a procedure similar to that used to validate the Level 1 subgroups.

### Construction of the structure similarity network

In analogy to sequence similarity networks, a structural similarity network for the TSF was created using the TM-align algorithm (64) to perform pairwise comparisons among a nonredundant set of TSF structures (File S1). The crystal structures used for input to TM-align were identified by searching the Protein Data Bank using Pfam (65) signatures PF14832, PF01187, PF08921, PF02962, PF01361, and PF14552. The resulting networks were then visualized in Cytoscape. A cutoff score of 0.5 or higher is considered statistically significant by the authors of the TM-align algorithm (66). A cutoff score of ≥0.8 was used for Fig. 8, well above the 0.5 cutoff score. To aid in comparing the topologies of the SSN and the structure similarity network, the 0.8 cutoff was chosen to match, to the extent possible, the subgroup boundaries used in visualizing the Level 1 SSN.

### Creation of HMMs

An MSA was generated for each named Level 1 and Level 2 subgroup using Clustal Omega (67) and manually refined. HMMs were created using HMMER 3 (68). These subgroup-specific HMMs were mapped to the relevant representative networks to enable a visual estimation of how well each captured the representative nodes of the subgroup and the degree to which each overlapped with other subgroups.

### Identification of TSF members without an N-terminal proline

Computational analysis of the 11,395 nonredundant sequences for those without an N-terminal Met-Pro yielded 2296 sequences. The 495 eukaryotic sequences were removed leaving 1801 prokaryotic sequences. Manual examination of these sequences for a correctly annotated start codon in-frame with a ribosome binding site resulted in the set of 346 sequences without an N-terminal proline. An explanation and examples of the manual curation process are provided in Fig. S15.

### Identification of linkers

Two edges were observed in the 50% representative network (Fig. 2) that connect the founder 4-OT and *cis*-CaaD subgroups. These two linking edges are defined by an almost identical mean BLAST *E*-value of 10^−11.015^ and 10^−11.111^, respectively. One sequence from each representative node connected by the edge defined by a geometric mean BLAST *E*-value of 10^−11.015^ was selected for experimental characterization. The representative node in the founder 4-OT subgroup contains only one sequence (Linker 2: UniProt F4GMX9). The representative node in the *cis*-CaaD subgroup contains three sequences. The best representative of these sequences (Linker 1: UniProt K9NIA5) was identified by creating an MSA and HMM for that subgroup and then selecting the sequence that scored highest against that HMM. Both proteins, designated respectively Linker 1 and Linker 2, were structurally characterized (see “Structure characterization” below). The representative nodes for the second edge connecting the 4-OT and *cis*-CaaD subgroups from the 50% network were named N1 and N2. N1 and N2 were included in the linker control experiment (Fig. S13), and N1 was included in the phylogenetic tree, as described below. Within the 4-OT and *cis*-CaaD subgroups, the nodes containing Fused 4-OT (PDB ID: 6BLM) and CgX (a *cis*-CaaD homolog from *Corynebacterium glutamicum* of unknown function (31); PDB ID: 3N4G) were identified as linkers that connect the representative nodes of Linker 2 and Linker 1 to those that contain the founder 4-OT (PDB ID: 1BJP) and *cis*-CaaD (PDB ID: 2FLZ) proteins, respectively.

### Creation of linker control networks

To determine the number of individual edges included in the representative nodes of the linker set, we constructed a 90% representative network (not shown) including all members of the *cis*-CaaD and 4-OT subgroups represented in Fig. 2. Twenty-five edges were observed that link 16 representative nodes in the founder 4-OT subgroup to 13 representative nodes in the *cis*-CaaD subgroup (generating a total of 29 representative linker nodes between the two subgroups at this threshold). The geometric mean BLAST *E*-value of these 25 representative edges ranged from 10^−11.015^ to 10^−13.046^. The first-neighbor nodes (one hop out) of these 29 representative nodes, along with their first-neighbor representative nodes of these representative nodes, were selected (in total, two hops out from the 29 linker representative nodes). A one-sequence-per-node network was then generated from the selected representative linker nodes composed of 2761 nonredundant sequences, as shown in Fig. S13. Forty-eight edges were observed that link 31 nodes from the founder 4-OT subgroup to 17 nodes in the *cis*-CaaD subgroup. The BLAST *E*-value of these 48 edges ranged from 10^−11.046^ to 10^−13.046^, indicating that multiple statistically significant edges link the 4-OT and *cis*-CaaD subgroups.

### Structure characterization

The details for the crystallization and structure determination of Fused 4-OT and Linker 2 (inactivated by the irreversible inhibitor, 2-oxo-3-pentynoate (25)) will be reported in a future publication. Briefly, crystals were obtained by the sitting drop vapor-diffusion method at room temperature. X-ray diffraction data were collected at the Advanced Light Source beamline 5.0.3 (ALS, Berkeley, CA) with a wavelength of 0.97741 Å (Fused 4-OT) or 0.97641 Å (Linker 2) at 100 K. Structures were determined using molecular replacement where the Linker 2 (inactivated by 2-oxo-3-pentynoate) structure (PDB ID: 5UNQ; 40% sequence identity) was used for Fused 4-OT and the 4-OT structure (PDB ID: 1BJP; 37 and 38% sequence identity with the N-terminal and C-terminal sequence, respectively) was used for Linker 2 inactivated by 2-oxo-3-pentynoate. The Linker 2 structure showed modified and unmodified active sites in different chains (of three chains/biological unit). The unmodified chain of Linker 2 was used in guiding the construction of the MSA shown in Fig. 5 as well as in the structure similarity network shown in Fig. 8. Both structures were deposited in the Protein Data Bank (Fused 4-OT, PDB ID: 6BLM; and Linker 2, PDB ID: 5UNQ).

### Creation of the structure-guided alignment

Chimera (69) was used to make a structure-guided MSA for the founder 4-OT and *cis*-CaaD sequences with the Fused 4-OT, CgX, and linker protein sequences. The structures used were founder 4-OT (PDB ID: 1BJP), Fused 4-OT (PDB ID: 6BLM), Linker 2 (PDB ID: 5UNQ), Linker 1 (PDB 5UIF), CgX (PDB ID: 3N4G), and *cis*-CaaD (PDB 2FLZ). The Chimera MatchMaker tool was used to align the structures, with CgX (PDB ID: 3N4G) as the reference structure.

### Phylogenetic tree construction of the 4-OT and cis-CaaD subgroups

As the time requirement for the computation of phylogenetic trees can become unrealistic for highly diverse sequence sets, the 90% representative network made up of 3006 representative nodes was pruned to provide a more realistic number of sequences for generating the tree. This was achieved by first generating a 70% identity representative network composed of 1496 representative nodes that included all sequences from the 4-OT and *cis*-CaaD subgroups. First-neighbor nodes (one hop out) of the 70% representative nodes containing Linker 1, Linker 2, and linkers N1 and N2 were selected, resulting in a total of 121 representative nodes. Subsequently, the sequence with the highest score to the associated HMM created for each respective representative node was selected for inclusion in the tree. From this set of 121 sequences, the 63 sequences composed of fused β-α-β-subdomains (sequence length >110) were selected to be used in the phylogenetic tree.

A structure-guided MSA of these 63 sequences was generated using Chimera. The Chimera MatchMaker tool was used to align the structures, with *cis*-CaaD (PDB ID: 2FLZ) as the reference structure. To create the MSA, the shortest path on the 90% representative network was calculated between the linker nodes of the 4-OT and *cis*-CaaD subgroups and the sequences representing Linker 1, Linker 2, or Fused 4-OT. That is, the subset of those 63 sequences that belonged to the *cis*-CaaD subgroup in the 90% representative network was aligned to a superposition of the structures of CgX and *cis*-CaaD. Similarly, the remainder of these 63 sequences, which belonged to the 4-OT subgroup, was aligned to a superposition of the structures of Fused 4-OT and Linker 2. Once this MSA was constructed, it was realigned using MUSCLE (70) to better resolve the gapped regions and alignment of variable regions among the sequences near the C termini.

This structure-guided MSA (Fig. S14) was used as input for computing the tree, which was calculated using the MrBayes software (71). The tree was calculated using one Metropolis-coupled Markov chain Monte Carlo (MCMCMC) chain from two runs with 1,000,000 generations sampled every 100 generations. The tree was visualized via FigTree using the radial layout (available from http://tree.bio.ed.ac.uk/software/figtree/).^5^

### Kinetic analysis of linkers

The substrates were prepared, and kinetic parameters were determined as described elsewhere (26). Nonlinear regression data analysis was performed using the program Grafit (Erithacus Software Ltd., Staines, UK). Linker 1, Linker 2, and Fused 4-OT were produced by tailoring a previous protocol as needed to obtain sufficient amounts of soluble protein at the purity required for kinetic measurements (26). The genes encoding Linker 1 from *Pseudomonas* sp. UW4 (UniProt K9NIA5), Linker 2 from *Pusillimonas* sp. (strain T7-7; UniProt F4GMX9), and Fused 4-OT from *Burkholderia lata* (strain ATCC 17760; UniProt Q392K7) were codon-optimized for their separate expression in *Escherichia coli*, synthesized, and cloned into the expression vector pJ411 by DNA2.0 (now ATUM, Newark, CA).

## Author contributions

P. C. B. conceived and coordinated the computational work, C. P. W. conceived and coordinated the experimental work, and both of them provided data analysis in their respective areas and wrote the paper. R. D. conceived computational approaches for analysis and comparison of superfamily data, developed and performed bioinformatics analyses, curated the superfamily in the SFLD, and wrote parts of the paper. B. B. generated the enzymes and carried out the kinetic analysis of Fused 4-OT, Linker 1, and Linker 2. B. B. also assisted in computational and phylogenetic analysis and wrote parts of the paper. J. A. L. and Y. J. Z. coordinated the crystallographic work and determined the X-ray structures of Fused 4-OT and Linker 2. C. R. P. assisted in the determination of the kinetic parameters in Table 1. E. A. provided expertise and guidance for phylogenetic inference and performed the analysis of the MIF one sequence per node subgroup. B. J. P. updated the SFLD backend as required for this project, generated networks for download from the SFLD, and contributed to the design of the linker control experiment. G. L. H. generated an initial set of TSF representative sequences, added them to the SFLD, and aided in the creation of subgroup HMMs.

## Supplementary Material

Supporting Information

## Footnotes

5 Please note that the JBC is not responsible for the long-term archiving and maintenance of this site or any other third party hosted site.

## Footnotes

4 The abbreviations used are:

TSF: tautomerase superfamily
CHMI: 5-(carboxymethyl)-2-hydroxymuconate isomerase
*cis*-CaaD and CaaD: *cis*- and *trans*-3-chloroacrylic acid dehalogenase, respectively
CgX: Cg10062 from *Corynebacterium glutamicum*
HMM: hidden Markov model
2-HM: 2-hydroxymuconate
MIF: macrophage migration inhibitory factor
MSAD: malonate semialdehyde decarboxylase
MSA: multiple sequence alignment
4-OT: 4-oxalocrotonate tautomerase
PPT: phenylpyruvate tautomerase
PDB: protein data bank
SSN: sequence similarity network
SFLD: Structure–Function Linkage Database.
