# Supplementary material for: A global view of structure–function relationships in the tautomerase superfamily
Source: J Biol Chem. 2017 Nov 28;293(7):2342–57. doi: 10.1074/jbc.M117.815340 (PMC5818174; doi:10.1074/jbc.M117.815340)
Supplement: Supporting Information [file supp_293_7_2342__index.html]

A global view of structure-function relationships in the tautomerase superfamily — A global view of structure–function relationships in the tautomerase superfamily — Tautomerase superfamily structure–function relationships — Supporting Information 

# A global view of structure–function relationships in the tautomerase superfamily

## Supporting Information

- Supporting info (.pdf, 3.8 MB) - Supporting info
