# Supplementary material for: A global view of structure–function relationships in the tautomerase superfamily
Source: J Biol Chem. 2017 Nov 28;293(7):2342–57. doi: 10.1074/jbc.M117.815340 (PMC5818174; doi:10.1074/jbc.M117.815340)
Supplement: Supporting Information [file 10.1074_M117.815340_jbc.M117.815340-1.pdf]

Supporting information for:

A Global View of Structure-Function Relationships in the Tautomerase Superfamly.

Rebecca Davidson[ǂ1], Bert-Jan Baas[§1], Eyal Akiva[ǂ], Gemma L. Holliday[ǂ], Benjamin J. Polacco[ǂ], Jake A. LeVieux[¶], Collin R. Pullara[§], Yan Jessie Zhang[¶‡], Christian P. Whitman[§2], and Patricia C. Babbitt[ǂ¥†2]

[ǂ]Departments of Bioengineering and Therapeutic Sciences, [¥]Pharmaceutical Chemistry, [†]Quantitative Biosciences Institute, University of California, San Francisco, CA 94143

[§]Division of Chemical Biology and Medicinal Chemistry, College of Pharmacy, [¶]Department of Molecular Biosciences, [‡]Institute for Cellular and Molecular Biology, University of Texas, Austin, TX 78712

Running title: Tautomerase Superfamily Structure-function Relationships

Table of contents

Figure S1. Oligomeric organization in the TSF……………………………………………………S3

Figure S2. Mapping of the Level 1 *cis*-CaaD HMM to the Level 1 *cis*-CaaD subgroup....................S5

Figure S3. Mapping of the Level 1 MSAD HMM to the Level 1 MSAD subgroup……………….……S6

Figure S4. Mapping of the Level 1 CHMI HMM to the Level 1 CHMI subgroup…………………S7

Figure S5. Mapping of the Level 1 MIF HMM to the Level 1 MIF subgroup………….....…………..S8

Figure S6. 90% sequence identity per node network of Level 2 subgroups of the Level 1 4-OT subgroup……………………………………………………………………………………..S9

Figure S7. HMM mapping of the Level 2 subgroup 1 to the Level 1 4-OT subgroup……….....……S10

Figure S8. HMM mapping of the Level 2 subgroup 2 to the Level 1 4-OT subgroup…………….…..S11

Figure S9. HMM mapping of the Level 2 subgroup 3 to the Level 1 4-OT subgroup………….....…S12

Figure S10. HMM mapping of the Level 2 subgroup 4 to the Level 1 4-OT subgroup…………….S13

Figure S11. Length histogram of 11,395 non-redundant protein sequences in the TSF…………...S14

Figure S12. Non-Pro-1 Frequencies in the TSF……………………………………………………S15

Figure S13. Linker control network…………………………………………………………......S16

Figure S14. MSA of sequences used to calculate the phylogenetic tree……………….……………S17

Figure S15. Examples of the curation process used to validate the non-Pro-1 sequences in the TSF………………………………………………………………...……………………..S20

File S1. PDB codes of structures used in the structure similarity network provided in Figure 8....S23

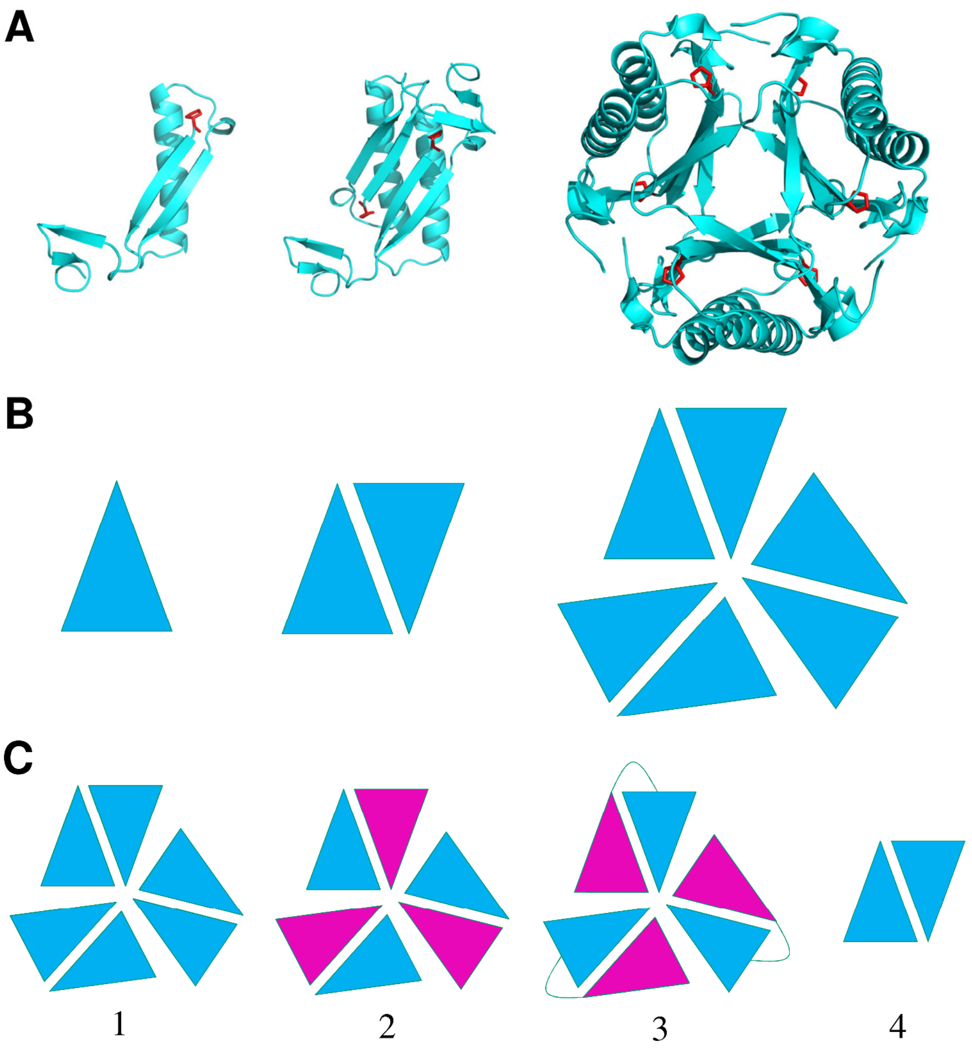

Figure S1. Oligomeric organization in the TSF. A. The three levels of structure for the founder 4-OT. The 62-amino acid monomer (left) showing the β-α-β-motif, the signature fold of the tautomerase superfamily. Monomers stack in an antiparallel fashion to yield a dimer (middle), which then make up the active homohexamer (right). B. Schematic representation of the same three structures. C. The four distinct patterns observed for the experimentally characterized members of the TSF. 1. Homohexamer, which includes the 'short' tautomerases such as the founder 4-OT. 2. Heterohexamer, which includes the hh4-OT and CaaD. Each heterohexamer is composed of three α,β dimers, where the α- and β-subunit fold into a β-α-β-motif, but are not

identical in sequence. 3. Trimer, which includes 'long' members of the *cis*-CaaD-, MIF-, MSAD- and CHMI- subgroups. Note that the monomers in each of these trimers are identical in sequence, but the two β-α-β-motifs within each monomer are not. 4. Homodimer, which includes YdcE, a 4-OT homolog from *E. coli*.

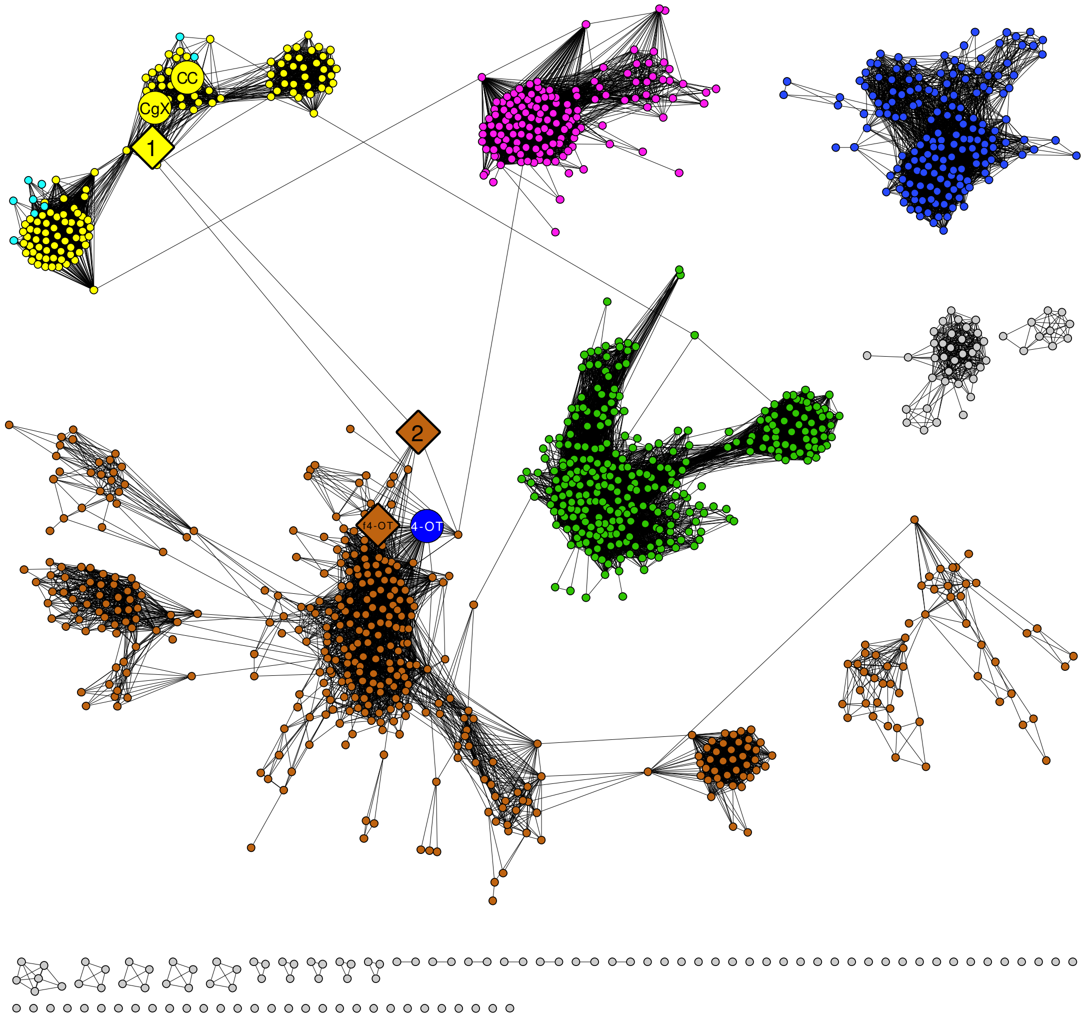

Figure S2. Mapping of the Level 1 *cis*-CaaD HMM to the Level 1 *cis*-CaaD subgroup. Details and color for this SSN are as in **Figure 2** except that yellow designates each representative node that contains at least one sequence for which its cognate HMM hits this subgroup at an HMM hit score of $1e^{-20}$. Nodes that retain the original cyan color of the *cis*-CaaD subgroup were missed by the HMM trained on this subgroup. The large labeled nodes indicate representative nodes that link the 4-OT and *cis*-CaaD subgroups as described in the section "*"Linkers" between* cis-*CaaD and 4-OT subgroup identify structural transitions between them."* The representative node of founder 4-OT was colored dark blue for consistency with **Figure 5**, and was not matched by any subgroup HMM other than that generated for the 4-OT level 2 subgroup 1 (see **Figure S7**).

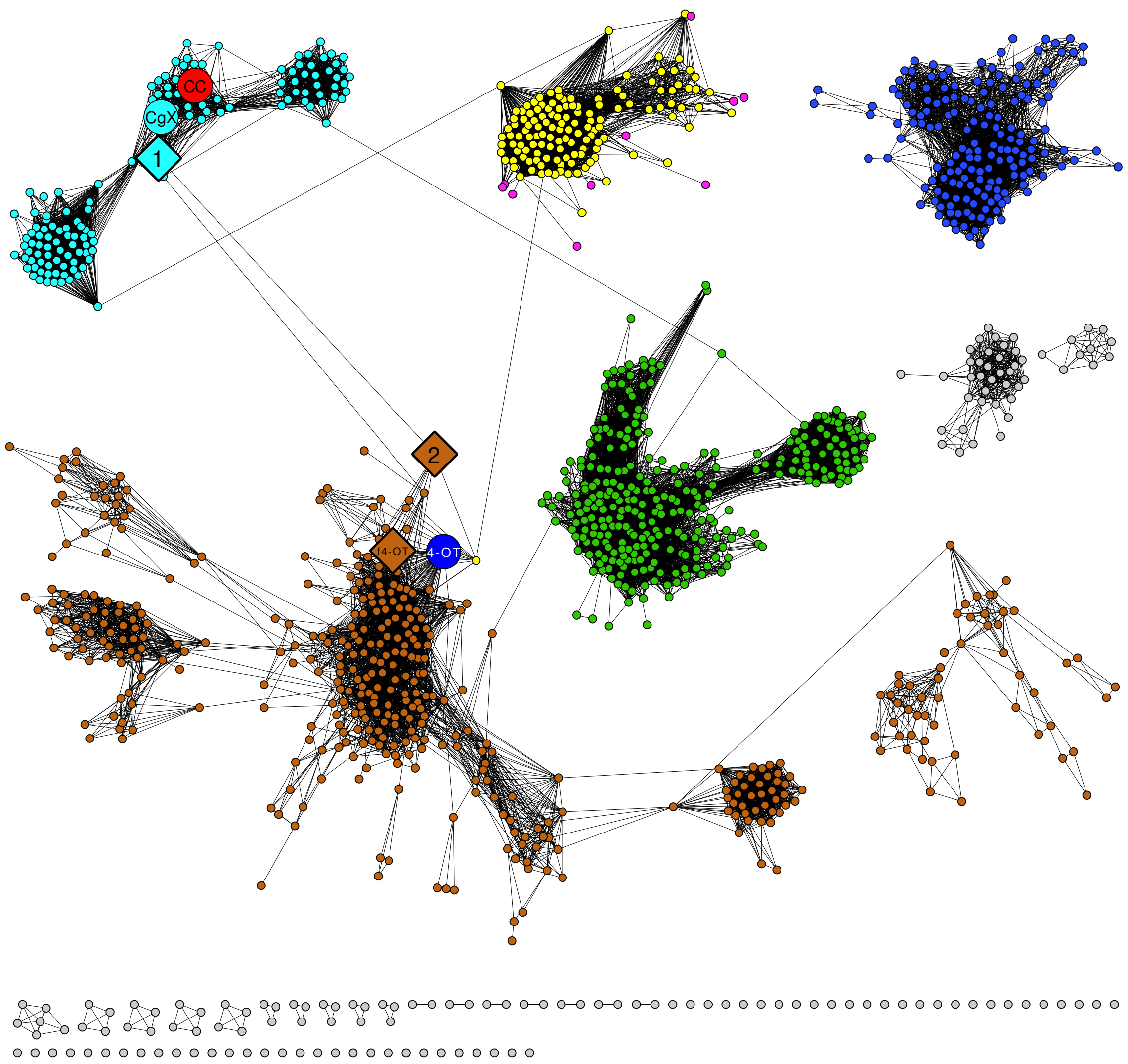

Figure S3. Mapping of the Level 1 MSAD HMM to the Level 1 MSAD subgroup. Details and color for this SSN are as in **Figure 2** except that yellow designates each representative node that contains at least one sequence for which its cognate HMM hits this subgroup or other subgroups at an HMM hit score of $1e^{-14}$. Nodes that retain the original magenta color of the MSAD subgroup were missed by the HMM trained on this subgroup. The large labeled nodes indicate representative nodes that link the 4-OT and *cis*-CaaD subgroups as described in the section "*"Linkers" between* cis-*CaaD and 4-OT subgroup identify structural transitions between them."* The representative node of founder 4-OT was colored dark blue for consistency with **Figure 5**, and was not matched by any subgroup HMM other than that generated for the 4-OT level 2 subgroup 1 (see **Figure S7**).

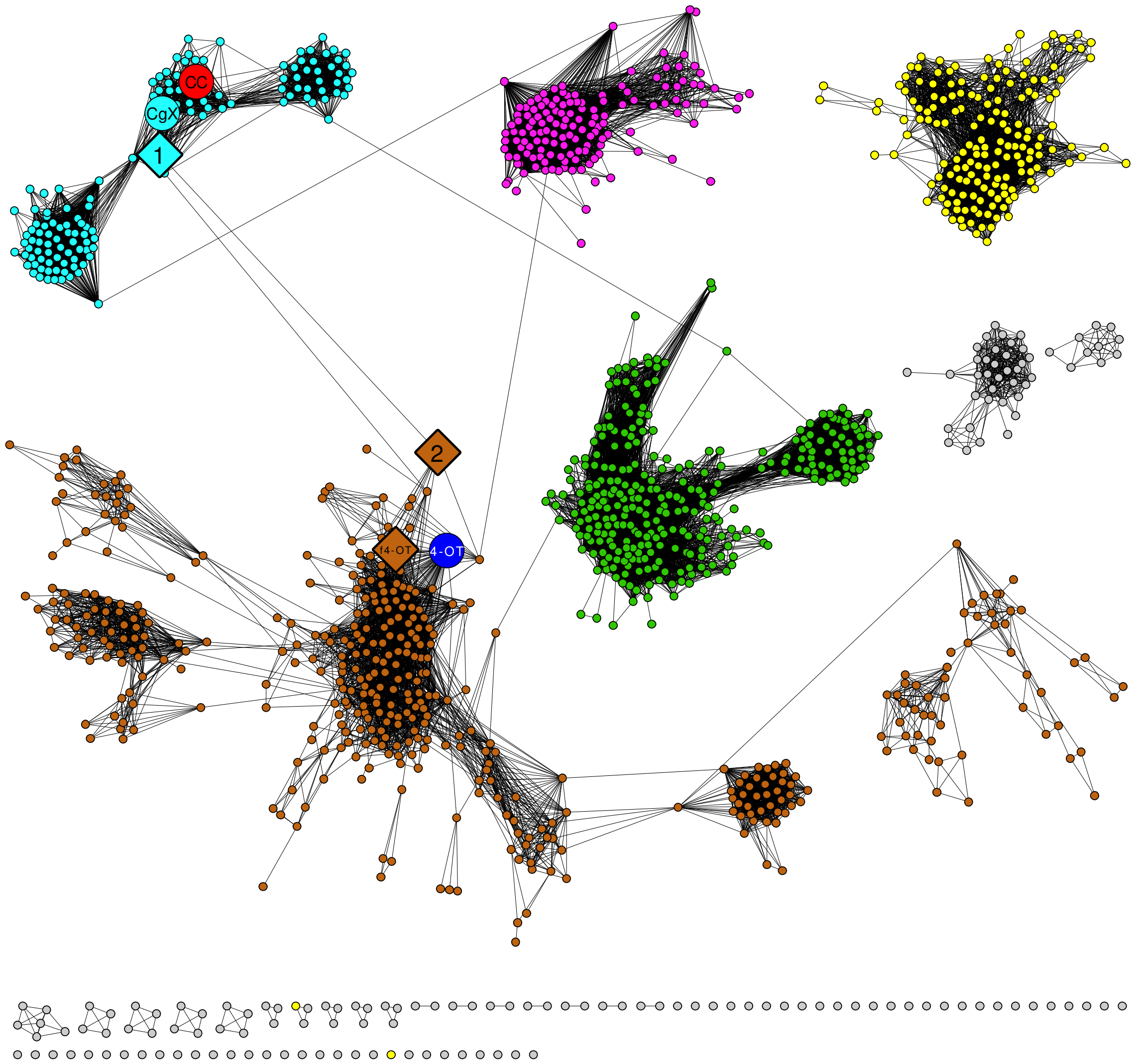

Figure S4. Mapping of the Level 1 CHMI HMM to the Level 1 CHMI subgroup. Details and color for this SSN are as in **Figure 2** except that yellow designates each representative node that contains at least one sequence for which its cognate HMM hits this subgroup at an HMM hit score of 1e^{-10}. Nodes that retain the original dark blue color of the CHMI subgroup (only the representative founder 4-OT node) were missed by the HMM trained on this subgroup. The large labeled nodes indicate representative nodes that link the 4-OT and *cis*-CaaD subgroups as described in the section "*"Linkers" between* cis-*CaaD and 4-OT subgroup identify structural transitions between them."* The representative node of founder 4-OT was colored dark blue for consistency with **Figure 5**, and was not matched by any subgroup HMM other than that generated for the 4-OT level 2 subgroup 1 (see **Figure S7**).

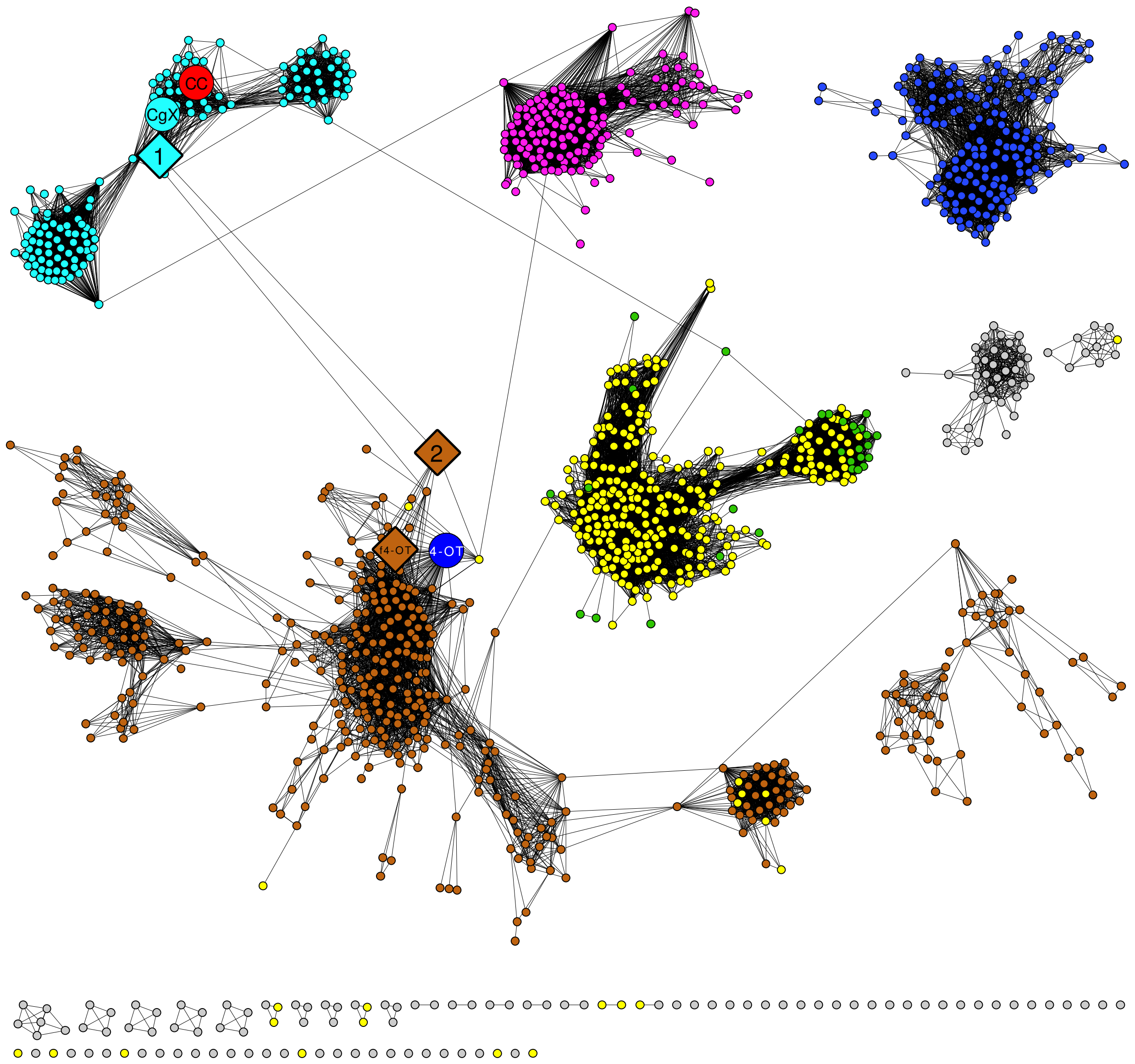

Figure S5. Mapping of the Level 1 MIF HMM to the Level 1 MIF subgroup. Details and color for this SSN are as in **Figure 2** except that yellow designates each representative node that contains at least one sequence for which its cognate HMM hits this subgroup or other subgroups at an HMM hit score of 1e^{-11}. Nodes that retain the original green color of the MIF subgroup were missed by the HMM trained on this subgroup. The large labeled nodes indicate representative nodes that link the 4-OT and *cis*-CaaD subgroups as described in the section "*"Linkers" between* cis-*CaaD and 4-OT subgroup identify structural transitions between them.*" The representative node of founder 4-OT was colored dark blue for

consistency with **Figure 5**, and was not matched by any subgroup HMM other than that generated for the 4-OT level 2 subgroup 1 (see **Figure S7**).

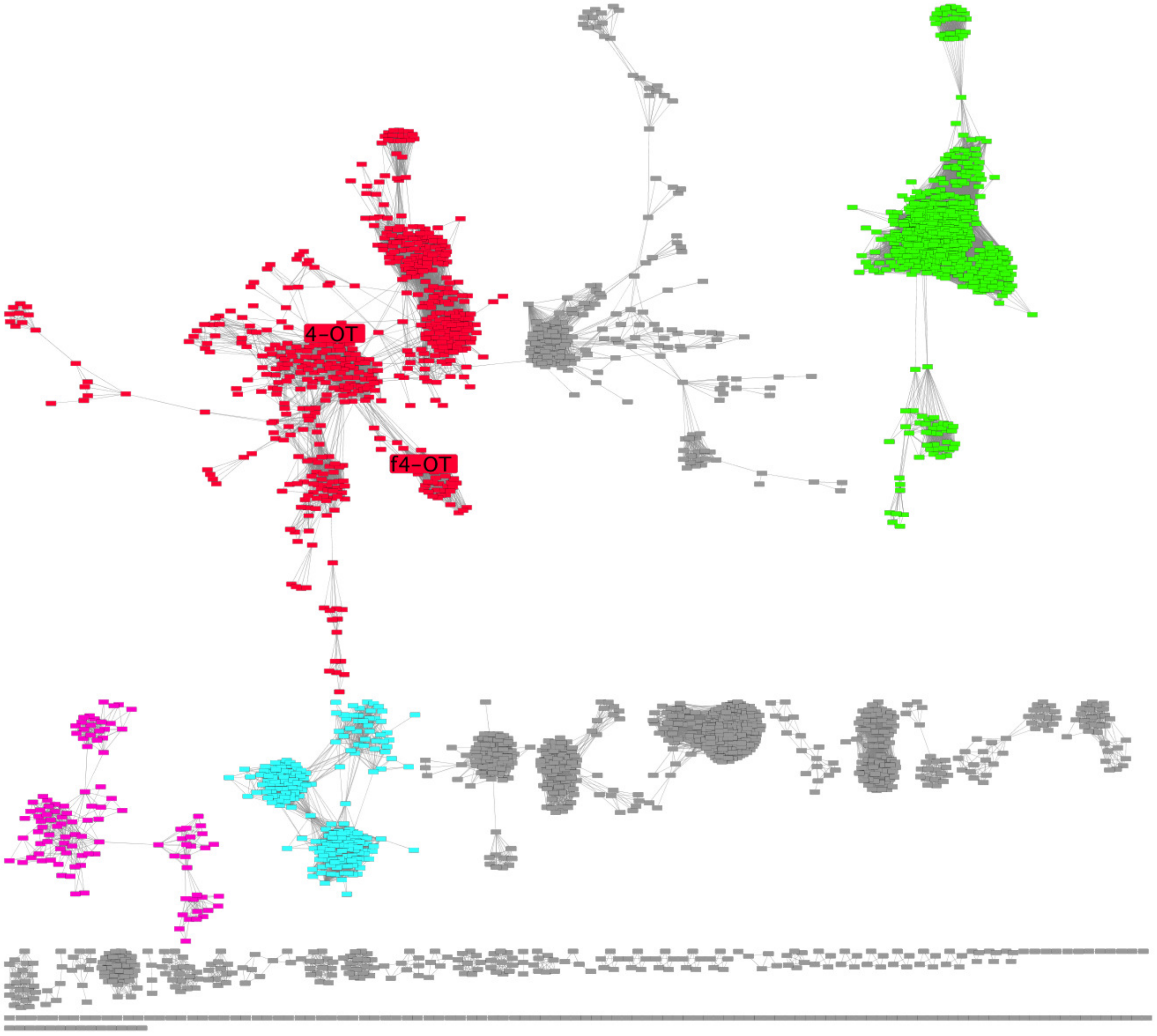

Figure S6. 90% sequence identity per node network of Level 2 subgroups of the Level 1 4-OT subgroup. 2,580 network nodes represent 4,530 4-OT like proteins colored by level-2 subgroups. The threshold for drawing edges between representative nodes is $1e^{-18}$. Gray nodes designate nodes that have not been assigned to a level-2 subgroup. HMMs have been generated for subgroup 1 (red), subgroup 2 (green), subgroup 3 (magenta) and subgroup 4 (cyan). The founder 4-OT and the linker Fused-4-OT proteins belong to the Level 2 subgroup 1, as indicated by the labeled large nodes.

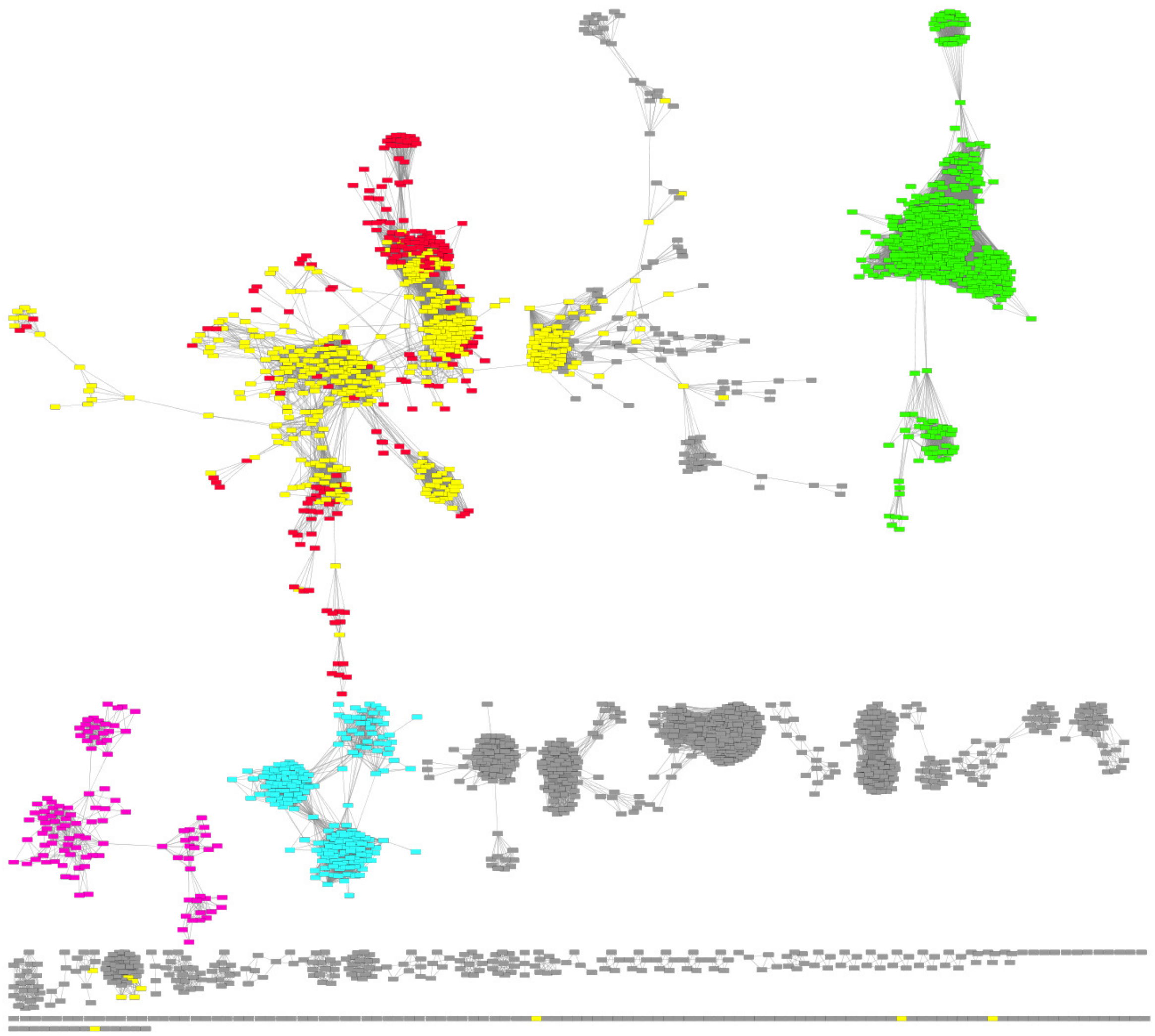

Figure S7. HMM mapping of the Level 2 subgroup 1 to the Level 1 4-OT subgroup. Details and color as in **Figure S6** except that yellow designates each representative node that contains at least one sequence for which its cognate HMM hits this subgroup at an HMM hit score of 1e^{-24}. Nodes that retain the original red color of Level 2 subgroup 1 were missed by the HMM trained on this subgroup. Yellow nodes that are not part of this subgroup are also matched by this HMM at the HMM hit score.

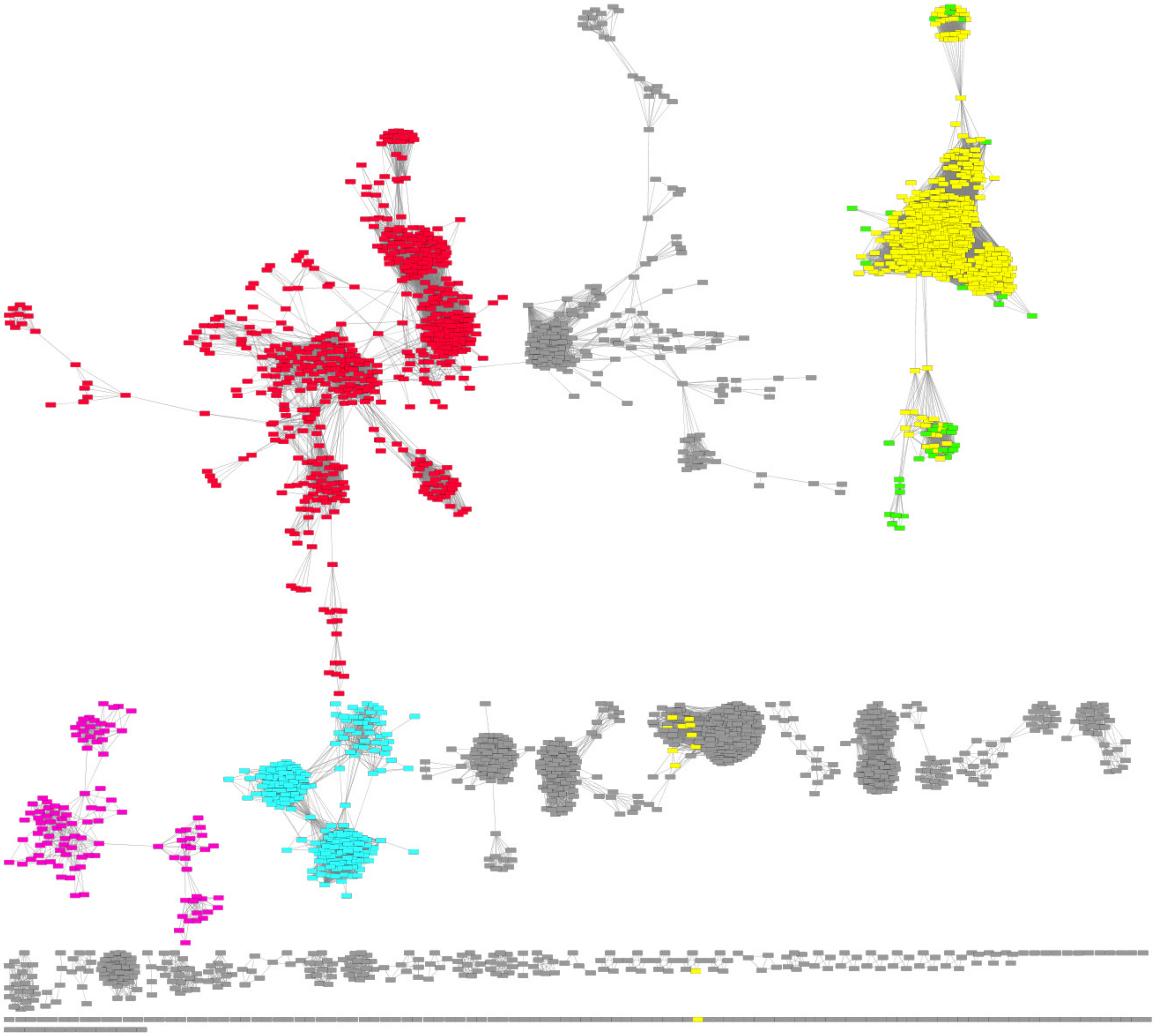

Figure S8. HMM mapping of the Level 2 subgroup 2 to the Level 1 4-OT subgroup. Details and color as in **Figure S6** except that yellow designates each representative node that contains at least one sequence for which its cognate HMM hits this subgroup with an HMM hit score of $1e^{-23}$. Nodes that retain the original green color of Level 2 subgroup 2 were missed by the HMM trained on this subgroup. Yellow nodes that are not part of this subgroup are also matched by this HMM at the HMM hit score.

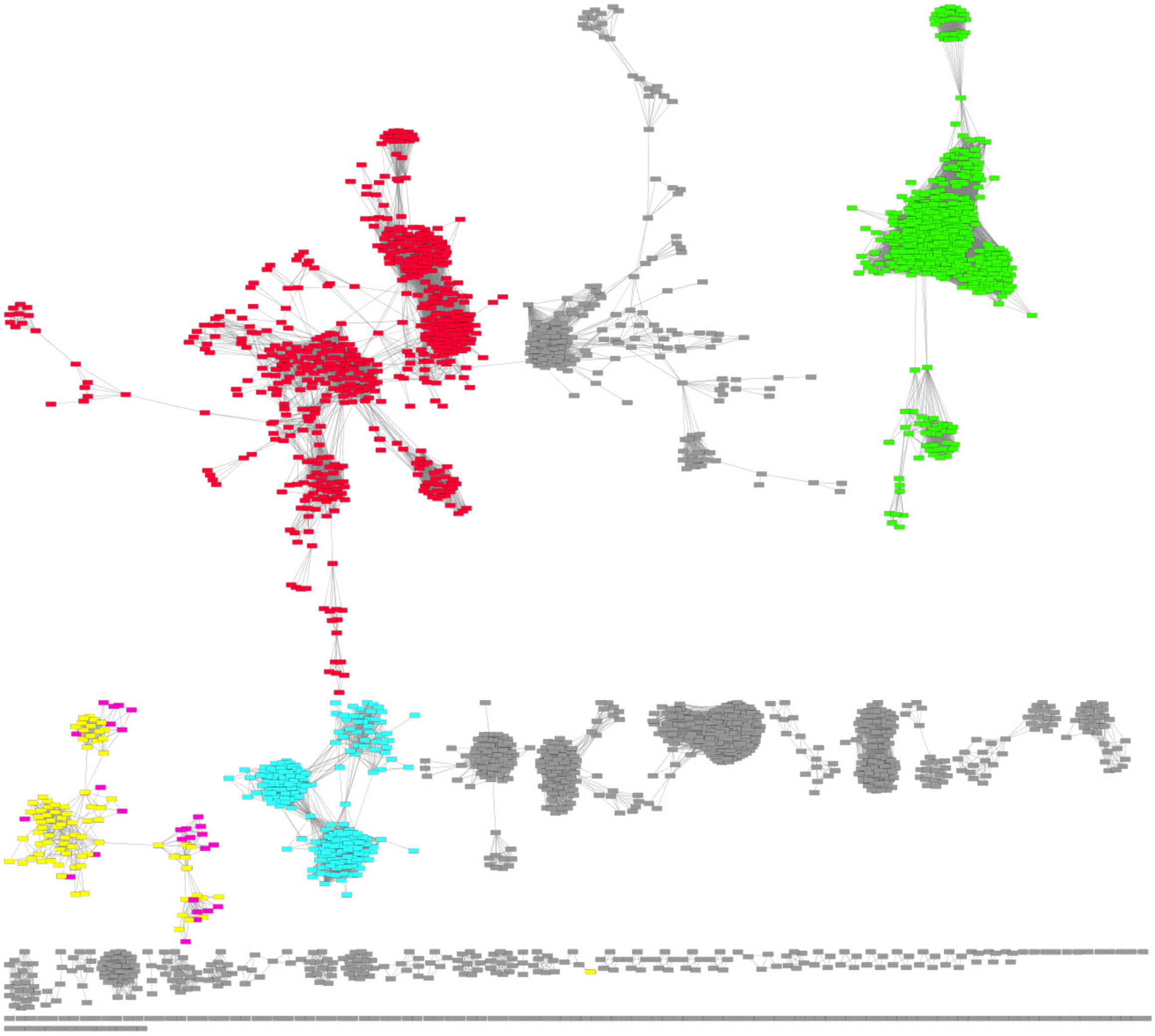

Figure S9. HMM mapping of the Level 2 subgroup 3 to the Level 1 4-OT subgroup. Details and color as in **Figure S6** except that yellow designates each representative node that contains at least one sequence for which its cognate HMM hits this with an HMM hit score of $1e^{-25}$. Nodes that retain the original magenta color of Level 2 subgroup 3 were missed by the HMM trained on this subgroup. Yellow nodes that are not part of this subgroup are matched by this HMM at the HMM hit score.

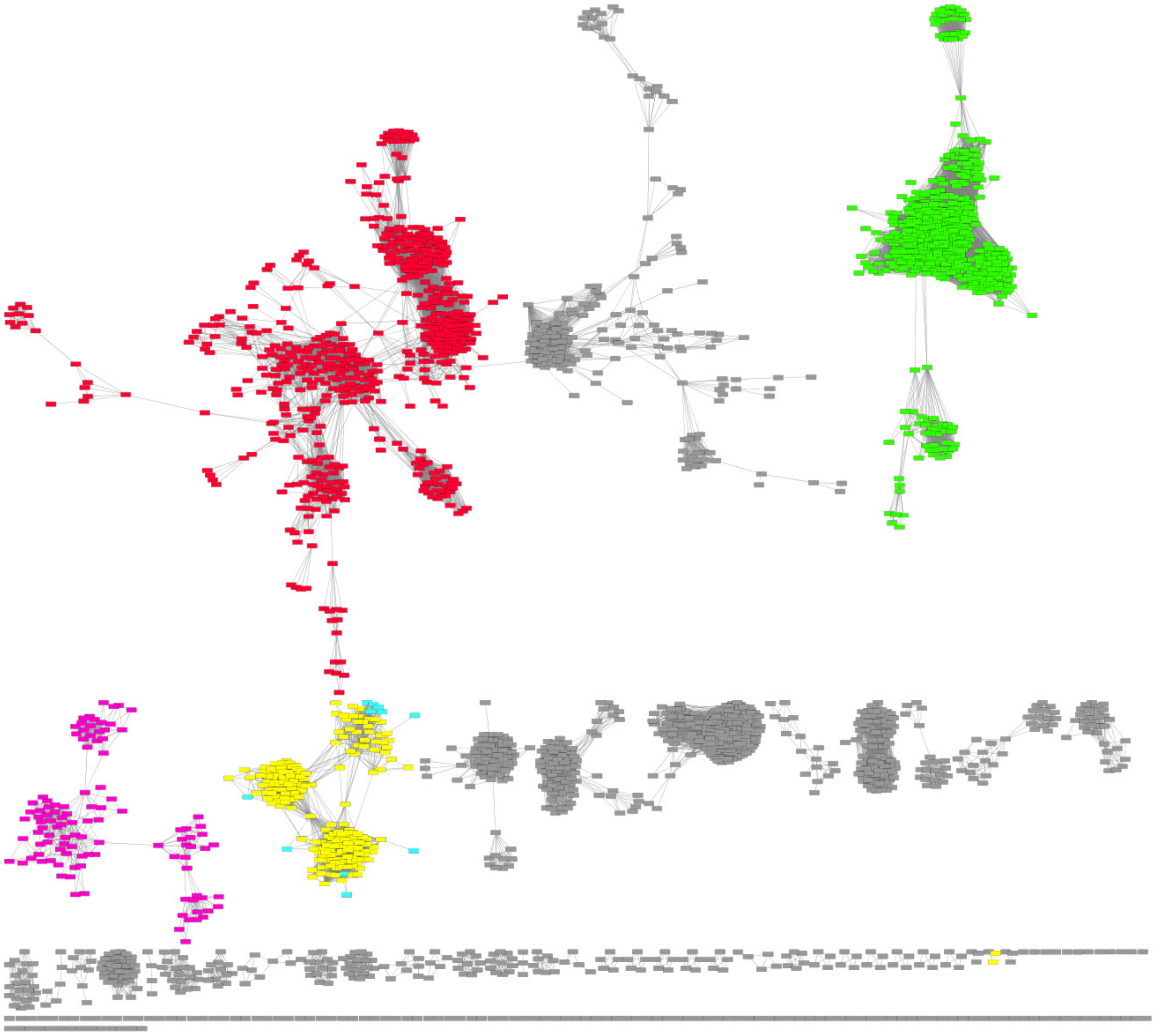

Figure S10. HMM mapping of the Level 2 subgroup 4 to the Level 1 4-OT subgroup. Details and color as in **Figure S6** except that yellow designates each representative node that contains at least one sequence for which its cognate HMM hits this subgroup with an HMM hit score of $1e^{-22}$. Nodes that retain the original cyan color of Level 2 subgroup 4 were missed by the HMM trained on this subgroup. Yellow nodes that are not part of this subgroup are also matched by this HMM at the HMM hit score.

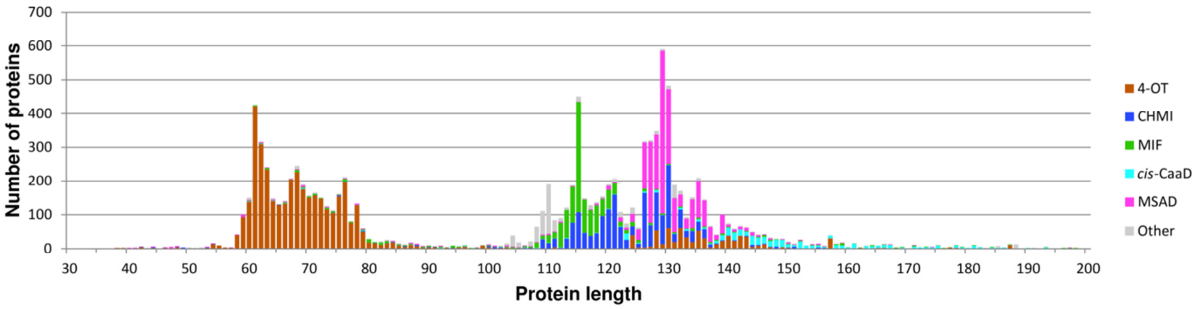

Figure S11. Length histogram of 11,395 non-redundant protein sequences in the TSF. The histogram is composed of stacked bars colored by subgroups as in **Figure 2**. A complex pattern in sequence lengths per subgroup is apparent. Sequences between 58-84 residues in length, which fold into a single β-α-β-unit, largely belong to the 4-OT-subgroup. Sequences between 110-150 residues in length are found in the other subgroups and fold into two fused β-α-β-units. There is “crosstalk” between these two populations, most notably the Fused 4-OTs. These proteins are part of the 4-OT subgroup in the SSN, but have a length similar to that of two fused β-α-β-units. The histogram shows sequences up to 200 residues in length, but there are longer members. These may represent proteins with a TSF-like domain, e.g. the 1357-residue indigoidine synthase IndC from *Streptomyces clavuligerus* that has a 76-residue C-terminal 4-OT-like domain.

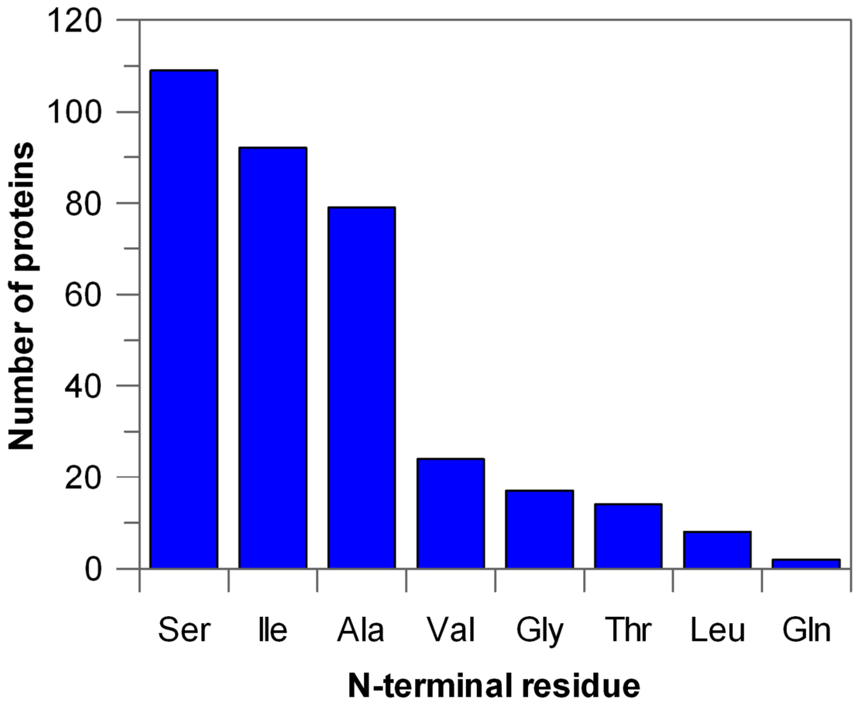

Figure S12. **Non-Pro-1 Frequencies in the TSF.** Histogram showing the frequency of residues in place of Pro-1 in the curated set of bacterial non-Pro-1 sequences (346 in total). Serine (109), isoleucine (92), alanine (80), valine (24), glycine (17), threonine (14), leucine (8), and glutamine (2).

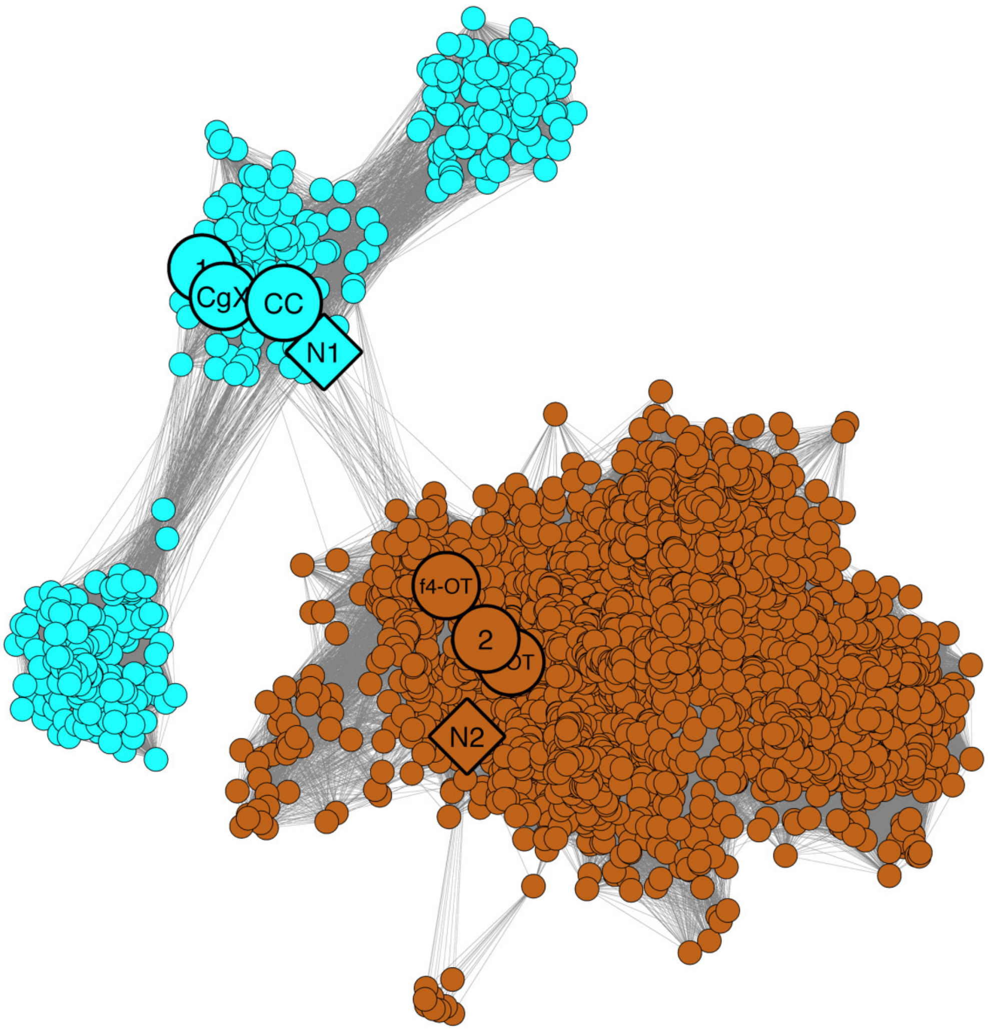

Figure S13. Linker control network. One sequence per node network of 2,761 non-redundant sequences expanded from the 90% representative network of the 4-OT and *cis*-CaaD subgroups. The threshold for drawing edges is $1e^{-11}$. The positions of the linker nodes are highlighted as shown in **Figure 5**, along with the positions of two additional linker sequences, N1 and N2. These latter nodes refer to the second pair of representative nodes besides Linker 1 and Linker 2 that link the 4-OT and *cis*-CaaD subgroups as shown in **Figure 5**. In this SSN, 48 edges link 31 nodes from the founder 4-OT subgroup to 17 nodes in the *cis*-CaaD subgroup.

Figure S14. **MSA of sequences used to calculate the phylogenetic tree.**

```
                             10        20        30        40        50        60        70
                    .    .    .    .    .    .    .    .    .    .    .    .    .    .
cis-CaaD/1-149      PVYMVYVSQDRLTPSAKHAVAKAITDAHRGLTGTQHFLAQVNFNEQPAGNVFLGGV------QQGG-DTI
CgX/1-148           PTYTCWSQRIRISREAKQRIAEAITDAHHELAHAPKYLVQVIFNEVEPDSYFIAAQ------SASE-NHI
Linker_1/2-113      PLYECQTVKGTLSERQRQSLAESITSIHTRETGAPASYVHVLFKELEPGSAFTAGQ------VATP---A
Linker_2/2-124      PYVTISATEG-LSAEKKKQLLERSSDAVVQSIGAPLASVRVMLHELPGGHYLNAGQ--------FNTPGL
Fused_4-OT/2-128    PTLEVFLPAG-HDDARKAELIARLTGATVDSIGAPIESVRVLLTELPATHIGLGGR---SAADGAPPSLP
A0A125QJV7/1-135    PLYICNAKAGAVPENAKAKIAEDVTRIHCEVTDAPPTFVHVF--------HFADGP-----MPPLGDKQV
A0A0Q6VD17/1-128    PLYSILTQAGMLDQNAKVELAEKLTALHCEYAGVPANWHIIFQDYRTGDGFIAGK------PAAT---T
A0A0C4YAG5/1-122    PVIVCECKVG-IEAGVKAKIASEFTSAIREIILSPLDLISVVFHESTPENTYRSGE------PTSE---T
X0QBL2/1-122        PVIQCHNRAG-LDLETKAQLAEEITRDVHEIVKSPNDLISVIFSDLPAESTYRNGQ------ATNE---T
A0A060ZNL3/1-122    PVIQCENRAG-FAPDVKAKLADEITAVVRDVIKSPMDLISVIFHDLPPESTYRSGA------PTDE---T
A0A149PHW3/1-122    PIIVCNTRAG-LDVDVKRKIAKAITIAVNETIKSPLEIISVVFNDLSSESSYVGGE------PGGD---T
A0A0Q7CP91/1-122    PLIMCYTRAG-LDVGTKAKLAAAITDKVHETIKSDFYHISVIFDDLASESSYVAGK------AGND---T
A0A149PTP3/1-123    PTLEVYLPQG-YADERKAQLIESLTEATVQAIGAPAESVRVLLSELGATNAGLGGK-------LTPAALP
A0A0S1Y1I0/1-123    PTIHAHITAG-PSAQQKSALLQAASQAVVESLGAPLRSVRLMLHDVAREDIVVGGE--------VGRESV
A0A157SPE8/1-123    PILNIQIMQG-HTDSEKTALLENATRAVEASIGAPVQSIRIVLEEVDARNVIVAGK--------LGHPMA
H0FC32/1-123        PILNVQILQG-HSAAQKAALLKAASNAVVESIAAPLPSVRIVLQEVPAEHVIVAGE--------IGKRMA
A0A158M531/1-123    PILNVQIMQG-YAPSAKTALLKSLSQAVVDSIGAPVASVRITLAEVDAGHVIVAGE--------LGKDMA
A0A063UWJ0/1-123    PLLNVQIMQG-HQPAQKSALLAAACQAVVDSIGAPLASVRVVLEEVPAAHVIVAGE--------LGRQMA
A0A142YAD3/1-125    PMYTIVIQSGSVDETAKASLAAEITALHVELSAVPKDWHVVFQEYAPGSGFNAGE--------AG-PLV
A0A0M4QA63/1-129    PYYRFTVPTGNATLAHRAEVAAAVTRVHSEVTGAPARYVHCTFIEVPPGSVFVAGE------PVEG---P
L8F8C4/1-130        PVYTCTTAVETLTPSSKAALATEITRIHSEVNHVPSTYINVVFQELPAEDVYTDAR------PAQP---L
B1FFZ6/1-127        PTLEVFLPAG-HDDARKAELIARLSGATVDAIGAPIESVRVLLTELPATHIGLGGR---TAADGAPPSLP
A0A0F5K0U6/1-130    PTLQIHLAQG-HDASQKAALIAALSHATVEALTVPIESVRIMIEDYAAIDHGAAGNAVPAAASRAEAPSP
L0IZU6/1-131        PIYTCTTTESTLTTDTRTALAGEITRLHSAINHVPSTYVNVVFHELPADAIYTDGR------PAAP---V
A0A101RJK5/1-132    PVYGVTTTRGIVSDEQKADLAAEITRIHSSVTGAPSSFVHVVFTELPEANVFTDSA------PSRP---L
A0A101QRI2/1-132    PIYTCTTAQGTLKAGAKGELAAEITRIHAEINHVPPAYVNVVFSELPQDSVYVGGE------PGAP---L
A0A0U3HBV1/1-132    PIYTCTCVQGSLPSDVKDGLAAEITRIHADINGVPPDYVNVVFTETPRENVYVGGR------PGTP---L
W9AW45/1-134        PVYTCTTVRSTLSADTKADLAAEVTRIHSMINHVPGTYVNVVFHELAPDEVYTGGH------PAQP---L
A0A0Q7FJ36/1-134    PTIEAFIAEG-YSHRQKHDLIEAMTHAVVDAIDAPIDSIRVILNEVATHDAGIGGK--------TGAAIS
D6TDA1/1-136        AVYTCITQEGALSAEQRADLAQEITRSHVAISGEPASFVRVIFETVSPNSTFSGGK------PAVN---A
R4X1K7/1-148        PTYHVSATEGLLDDKARARLANEITRAHSLATGAQSFFAQVLFHETPKRFHFMGGK------PVDS-EQV
A0QSU8/1-138        PVYTVTMSRGTLNGETKAALAAEITTIHSAVNHVPSTYVNVLFNELAPSNVYTDGK------PAHP---L
W4JVR2/1-138        PLYVLYYHAGSLSFEQKQTIAKEVTDAHCAVTGAGPYFVKVAFQACERGDFYTAGD------IDQ--KHV
A0A094IHN9/1-139    PTFVVTATAGCLSADKKAAIANALVTIHSTENGVPRWCVQVIFHEISAGNHFIGDK------FAPA-DQL
A0A0R3F487/1-141    PLYQCITRAESLTDEVRQTIAQEFTRIHCEITGAPAVFVHVVFNEYQPGHHYLAGK------PES--DTT
I2AHZ4/1-141        PVYQCYSPPGLLSESAKARIADEITTIHTSATGAPELFVNVLFHEIPVGDCFVGRK---------QASHS
A0A0P8AZP5/1-142    PTYTVTVANLSLSAEQKSQIAEAITAAHNAQTGAPRFFAQVLFYAANEGDHFVGGR------VNTA-PQV
A0A0D6IB59/1-142    PLYQIDTVKGRLTPSVKAEIANKVTDIHCQLTGAPDTFVNVVFREYTEGDCFVARK------PEGR---S
W9GWJ5/1-143        PTYAFSTAKE-LTAEQRAKLVESVTSIHQVEAAAPRYFVQVIFHKIEPGSMFIGGE------AASP-DHV
A0A0M2XHR9/1-143    PTYTVRTSTGRLGPDARASLAHAITSAHTVATGAPGFFAQVVFEEVDTDRHFVGGT------PIAD-ELV
A0A0S2PHN2/1-143    PIYQCSAPVGLLTDDMKAAVATAITDAHVEATGAPKAFVHVFFHELPPGIAYSAGE--------LDTDIS
A0A0E4GVS2/1-143    PVYQCVSSAGLVSADARARIAQDITRLHCEVTGAPAAFVNVLFSEYVSGELFTGGQ------PSQN---S
M1NLA4/1-144        PSYAVSSRAGLIDQERRAAVADLLTTLHRDIAVAPRYLVQVIFNDLDAGALFLAGR------EAPE-GHV
R7XJL4/1-144        PTYVCWTKTGRLSSEQRAQIAKSVTEIHHEVGRAPRYFVQVIFNELGAQSHFIGGT------EASV-DQI
A0A0Q5VHB9/1-144    PTYTCWSESGLVGPEQKQAVATALTEIHHEVAVAPRYFVQVIFTELVPGSVFLAGQ------PATR-GHV
A0A0T1WIY8/1-145    PTYTCWSKTGVVATEARARIAAALTRIHYDVAAGPRYFVQVIFTELDPDSLFIGGK------PVTA-EHV
A0A0P9CHA8/1-145    PTYILKSSKLKINKTKRDLLAKGITRIHSKVTGANSYFAQVIFQENINGSHYMGGK------PVQS-KEV
A0A0B6S9Z8/1-145    PTYVVSAAAGRFSQEEKEGIARGITQAHGRATGAQGFFAQVIFHWIAADDHFVGGR------VHDA-DHV
W6X611/1-145        PTYTVMAAAGRLTDPQKREIARDITRVHSEATGAQGFFAQVIFQAIPAGDHFLGGA------PLVS-DQL
F2LFU5/1-145        PTYVVSAAAGRLDAQQKARIAAGITEAHSRATGAQGFFAQVIFVPIAEGDHFLGGR------PLKS-DQI
A0A0Q7D9D7/1-146    PTYVVTAPQGRLSAQQKERIGADITRVHCTVASAPAYFAQVIFNDVPAGNYFVGGK------LLQRSDHV
A0A077K980/1-147    PTYIVSATQNLLSPEEKKKVAKEVTRAHSQATGAQGFFAQVIFSEIPSGSHFMGGV------EISA-KQI
A0A126YPH4/1-147    PTYIVSTAADRFNDELKHRIADGITKAHSRATGAQGFFAQVIFNEIPAGNHFIGGS------PLKA-EQA
I3U9B8/1-127        PILQVQVTAG-RSQQQKTAFLQNATKVIEQTLNAALPSIRISLQEVEQQDSIVAGQ--------IGAEFV
K8P2T6/1-148        PTYTVTTANLSLSAAQKGQIAEAITAAHHAATGAPAFFAQVIFRALENGEHFIGGK------PNAH-PHV
E6W3C6/1-148        PTYTVTAPSGRLNAEQKQNLATAITRAHHDITGAPTYFAQVIFVEVQPGNYFVGGA------PLAH-DQI
A0A0D1NR67/1-148    PTYTCTSAEGRLSAEQKSRIASEITRIHAEVTGAPSYFAQVIFSEVASGNWFMGGV------PVAH-DHI
A0A0S7Z4Z0/1-149    PTYHCSARMGLLDAERKARIARAVTLAHAEVTGAPPHLAQVLFRDVPPQDHYVGGA------LLEH-DHV
A0A0G9H7B6/1-149    PTYIVRSTLPSLTAPTKQRIAQAITAAHADITGANTFFAQVVFDHAPGDDWFIGGV------PVED-ATL
A0A089UPG7/1-151    PTYYCHLPKNRVSDVDKHRLAHAITARHTEATGAPSWFVQVVIEEDDDKIRYLGGE------PAGE--HI
A0A085G1G7/1-151    PSYVCSVPNGLLSDAQKQEIATCITHSHCEATGAPPFFVQVIIEEEGTLKRYIGAL------ATTE--YI
A0A0D6P3I9/1-162    PTYECRAPAGLLDAARRARIAAAVTRIHRQVTGASAYFAQVIFTEVADGAWFLGGT------PLVG-PHL
A0A0E9NHE3/1-135    PLYTLYYPPGAFTQWEKPAIAKRITDVHSSVTGAGAFFVKVIFVPVPAGDIFSGGK--------LDDQLV
```

```
70        80        90        100       110       120       130       140
IFVHGLHREGRSADLKGQLAQRIVDDVSVAAEIDRKHI--WVYFGEMPAQQM-VEYGRFLPQPG--HEGEWFDNLSSDER
IWVQATIRSGRTEKQKEELLLRLTQEIALILGIPNEEV--WVYITEIPGSNM-TEYGRLLMEPG--EEEKWFNSLPEGLR
AIIRGQIRAGRPQATRHAILRAITDVYMAVTGADANAV--VVAVVDIPASWA-ME-------------------------
LMFVVDFIEGRTEEQRNALIAALSKTGTETTGIPESDV--RVRLLDFPKANMGMAGGISAKAMG--R-------------
PVIVAILIAGRTDEQKRALIAALSETSASVLDAPLQAT--RVMIKDIPNTDF-GIGGQTARALG--R-------------
VMLYGQIRHGRTDDQKAEIVSQMQASVVEHTGLAPEAV--HVFTTDTPASWV-MEGGDILPEPG--EEDEWLERHNAKNH
TSLTLLIRTGRTADYKRGLLTRLWALVQSATGASDEEI--VLGIHEVPPSQA-MEMGKLMPNVS--ESPADTQ-------
TLIFCHIRDGRSDGAVLSLAKKVSTIWSACTGATEDEV--EVLVTLYPAKYV-VRGGERLPEAP--RV------------
TVIFCHIRKGRTDGAIERLLKTISHTYAKFTGLGLDEI--EVAAAEYPAVHT-MRNGQLLPEPP--IV------------
TLIFAHIRAGRSDEAIQSLLKSISEAWSRITGDSEDNI--ELAVQQYPAKFT-MRGGRRLPEPP--IV------------
TLIMCNIRAGRSDEAKLTLVKKVSAIFSDYAGVSEDRI--ETGLLEFNPKFI-IRGGQQLPDPP--YA------------
TLIICNIRVGRSDGAIQKLSKAISDIWHDITQQSEDEI--EVAVQEFQGKFV-VRGGKPMPEAP--YA------------
PVAIAILIAGRTDAQKVALIAQLSDAMSAILDVPLPST--RVMIKDIPNTDF-GIGGKTARALG--R-------------
VVFHVHMIVGRSEDQKQALFTALTKAASSTLGVDGENV--RVIVQDVPNTDMGMANGVSAKNTG--R-------------
ALALVRLIAGRDEAKKATLIAALSLAIHASLGIAEQDI--RVVLTDVPNTDMGVAGGLTAKAAG--R-------------
ARVDVELIEGRDEAKKAALIAALNQAVCASIDISGEDV--RVLLRDVPKTDMGVANGLSAKAAG--R-------------
ALIIVDLIAGRTPELKSALISALNQAACESLGISGQDV--RVVLHDVPKTDMGVANGLSAAAAG--R-------------
ALVTVDMIAGRTDEQKEALIGALNRAVCDSIGIDGTDV--RVMIHDVSKANMGVANGISARAAG--R-------------
VALTAAIRSGRSADYKHRLLTSLWTLVKGATGALDEQI--VVGLQEVGPGQA-MEMGRVMPEVD--SNVS----------
PRMVGLIRDGRTAEVRARLLHGIADAWCAVTGDAKADV--AIFLHEVPGANV-LEDGEILPEAA--DDPVAAHG------
LIIRGWVRSGHPEDETSQLVAQVAAAATAVTGIPKERV--LVIIENSPARFA-IEGGRVLPEPG--QERAWLEAH-----
PVIVAILIAGRTDEQKRALIAALSDAGANVLDAPLQAT--RVIIKDIPNTDF-GIGGQTARALG--R-------------
PIVFALLIAGRTAAQKAALIAQIDRACVTVLGATREPS--RIFIKDIPNTDF-GLAGQTARSLG--R-------------
VLVSGWTRAGHPDAETTRLATEIAAAVTRIAGIPAERV--MVVFVSSPAHYA-VEGGRVLPEPG--HEQAWIAGSG----
LLIHGTTRAGRPDAEKVRLAKSISTASSEITGVPESRV--LVIITDTPARFA-VEGGRVLPEPG--DEDDWLDEQSN---
LLISGWARRGHPQEETTRLALELSAAASRITGIPERRI--LVVIQDSPARSA-VEGGQVLPDPG--QEKEWLSRHEA---
LLINGWARRGHPQDSTTRLALAIAEAASRISGIPQEHV--MVVILDSPARSA-VEAGRVLPDPG--HEAEWLAAGRS---
LLINGWVRTGHPEAQSSQLVAEIAAAATRVTGVPAERV--LVVIQNSPAHFA-IEGGRVLPAPG--EEEAWLREQKDTD-
SVAQAFLIAGRSIEQKRRLIAGLTVVMAALPGVDSGGV--RIIIKDIPNTDF-GIAGQTAQSLGRGIDRSAMAAAAR---
ASIVGIIRS-RSTEVKAQLLNDLWSMFKNVTGLSDDQL--WVSVTEIPPSNA-MEFGAIIPEVG--REAEWQASLGLTKE
VFVHGHIRSGRTTDQKVKLLGDILGSVRQVTGLDSRYV--WIYLSELPPSDM-IEYGQVLPQPG--AESAWLQALSEEDR
LIINGWVRTGHSDEQTTALVTQVADAATRITGIPAERV--LVIIGNSPARFA-IEGGRILPDPG--QELAWLAATTEQSS
VRFVGYVRRGRNPARMQELLQRLYRSVR--VGQPDETVDIEMHVQEADADVW-TLNGVNLPQPGSDEEQRWNTACGVPKQ
LWIHADTRPGRTTEKKTAMIDRMVKEVSTAGGVDESYI--WVYVNEI--SEM-AEFGMHFPPPG--MEGAFVASLPVEVR
TFIAGTIRSGRSLEQRQQLLRELSSAWHELTGQPEEEL--VLSISEQDASAV-MEAGLIFPEAG--AEAAWFEQNREKLG
SYLFGAIRHGRDAATRQTMLREFSRMWSRATGQSEAEF--LVALTEVDPANA-MEAGLVLPEPG--REQEWFDENRVRLT
VYVHGLVREGRSIEIKQALMSQMLEEIAQIVDITAEDV--WIYLQDIPATQM-IEFGRFLPAPG--GEAEWEKGMTPEKH
SFLGGQIRHGRSVETRQAMLKALRDMWVQTTGQSEAEL--IVGISEVDPRMV-LEAGFFMPEPG--QEKAWFEEHHARLA
VWVRADIRSGRTNEQKANILRRVMRETSEILGIAEQAV--WVYISDIPAQGV-LEFGNVLPEPG--GEEQWLASLPSALR
VFVHGQIRAGRTPEQKVALLDALSGVVREVLGVPRRTV--WVYLVDLPPADM-IEYGYVLPAAG--EEAEWLASLDDDTR
SGITGSIRAGRTLEVKQKLVKDIAASWTSITGQSPKQL--IVGLNEIDSDIT-MEYGLILPHPG--GEAEWFATHADELD
SFIAAEIRAGRDLDTRQALLRELSGIWTEATGQNEAQL--LVAIKETPAENA-MEAGLMFPKPG--EEAQWMSENSDKLA
VWIHADIRSGRTAQQKTDLLEQITSKVADVLELPPEHV--WVYVNEIPGENM-TEYGKLLPEPG--KEEEWFATLPQSLQ
IWIRADIRSGRTQEQKDQLLMRIADEVSEIAGTSKESV--WVYISDIPGPSV-LEFGRILPPPG--EEDTWFAALSVELQ
VWIRADIRAGRTVEQKRALLERITVEVGAILGLPPEEV--WVYVCDIPGSSI-AEHGRVLPEPG--GEDAWFDALPPDLQ
VWIRADIRAGRTEETKAELLRQIVAEVGDITGVVAEHV--WVYLNDVPGPNI-AEYGRPLPNPG--QEDRWFEQLPRELQ
VFLHGHIRAGRTNVIKKKLIEKLRDSIKKDLNLSKDQV--WVYISELEPSQM-IEYGEILPKSG--QEKKWFNQLPKKLK
VTVHGTIRAGRTAEQKSRLLEDIVGIVAGAANTGRRHV--WVYLAELPPAQM-AEYGRVLPQPG--GEAGWLEALPAEDR
LFVHGQIRAGRSADQKRDLLEALVTLVANATGAEKRSV--WVYLSELPPSQM-VEYGKVLPEPG--AESEWLNSMPEPDR
IHVNGQIRAGRNAEQKRRLLDAIVELVTRAAQAEPRAV--WVYIADVPPSQM-VEYGRVLPEPG--EEANWLQAMPDEDR
VYVHGQIRAGRDGETKERLVLELMNAVADAAEMPAHCV--QIYVVDVPARQI-AEYGQLLPLPG--GEAAWVAAIPAELR
IFVHGHIRAGRTEEQKGALLADIINSIHGVTGIEKRFL--WAYISELAPNNM-VEYGQVLPQPG--KEAEWLESLSAADR
AIVHGHIRAGRTPEQKRQLLESIVEVIISATALERRYV--WAYISELPPSQM-VEYGRVLPEPG--AEEDWLKSMDEADR
VNIVAFLLAGRNDELKADFMAAINKAAVTSLDVSDTCI--RTMVIDVAPEHMGVQEGLSAKAFR--ARSAS---------
VFVHGMIRSGRGIDVKQKLMGEAADKVRASAGVGAEDV--WFYIQDIDAPQM-IEFGRFLPEPG--AEDAWRKAITPEKQ
IFIHGQIRAGRSEEDKRKLIEKILLVCSPAAAAPASAL--WVYLVDLPAAQM-AEFGHILPEPG--QEAQWSASLPAADR
IFVYGHIRSGRAAVDKTRMIRLMADAIRAAANVDSTRAV-WVYLNELQPRQM-IEFGHVLPEPG--DEPAWTEALPDADR
VFVHGHIRGGRSTELRAALVRRLTDDVAATARVDRLAV--WVYLSEIPAAAM-VEFGHVLPEAG--QEAAWTEALPAADR
LFVHGHVRSGRTDHQKRMLVERLVRDVAEASGLPTRAI--WIYLSEIRPSLM-AEFGHVLPEPG--EEAAWFDALPEDDR
IWVRADIRAGRTKSQLQQLMLALKTDIASITDVAAEDI--WIYLNNIEPDNM-LEYGHVLPLPG--EEKRWFDALPAELQ
IWIRADLRAGRSEEQLKALMLRIVGDVSRISGIPAENV--WINMCMLNPTDM-VEYGRVLPGPG--EEQQWFETLPQPLR
LFLHGHIRAGRSAGDRMRLIRRLVAALAEGASLPPRSV--WVYVTELPPRAM-AEYGHTLPEPG--DEAAWFAALPPEDQ
VRITGVIRAGRDTEARQKILRGVWEGMQEFLGERKC----EMHLEEMLGENVIAENGKFMPESGTDEEKRWNGNGGPM--
```

```
150       160       170
AFMETNVDVSRT-------------
ERLTELEGSSE--------------
-------------------------
-------------------------
-------------------------
AKAK---------------------
-------------------------
-------------------------
-------------------------
-------------------------
-------------------------
-------------------------
-------------------------
-------------------------
-------------------------
-------------------------
-------------------------
-------------------------
-------------------------
-------------------------
-------------------------
-------------------------
-------------------------
-------------------------
-------------------------
-------------------------
-------------------------
-------------------------
-------------------------
EA-----------------------
AYLNRLAERAE--------------
GSR----------------------
-------------------------
QRYE---------------------
FLLNQ--------------------
QMGMAL-------------------
GSLPK--------------------
ELGVQAG------------------
EKLMRSA------------------
SYLAPD-------------------
GIQGTGL------------------
ALGWNVST-----------------
EELSDLR------------------
ERLRSLT------------------
ERLAALR------------------
ERLRTIAE-----------------
NKLLKLDF-----------------
DYLLGLDK-----------------
KYLLDIGK-----------------
AHLVGIGQ-----------------
KHMEAIGE-----------------
DYMLSLSKAQ---------------
DYLLGMGAGS---------------
-------------------------
SQLGKAGVRVL--------------
QRMEAVGKTAQ--------------
SFMQSIGKRR---------------
EHMEAAAARFRR-------------
RRLAAIAERARS-------------
HRLEALGVTHANFTL----------
ERLATLGIDQHKFQL----------
AHLRALGAGDAGDAGDDGAGPGTAN
-------------------------
```

Figure S15. Examples of the curation process used to validate the non-Pro-1 sequences in the TSF.

As it was surprising that multiple sequences lack an N-terminal proline and are represented globally across the superfamily, it was important to validate this result in detail. Initial bioinformatics identified 1,801 non-eukaryotic sequences without an N-terminal proline. (Eukaryotic sequences were not examined due to the complex nature of their splicing maturation.) Each nucleotide sequence was examined manually and most were removed from the set due to misannotation of the position of the initiating Met or other technical issues (see below). Ultimately, 346 sequences were validated as missing an N-terminal proline.

An example of a properly annotated sequence missing Pro-1 is shown in A).

E2PB85	MISVYGLKQTLADRRALIADVIFDCMQMSLGVPKQRHALRFDLLDAENFYPPINRSQDFI
W0R8J4	MISVYGLKHTLAPRRALIAEIIFSCLEVNLGIPKQRHALRFELLDDENFYLPVNRSDNFL
I3DIP6	MITVYGLKKSLAPYRKQIADAIFHCLHIGLGIPPRKHTLRFVGLEKEDFYLPINRTERFI
I3DR40	MITVFGLKSKLAPRREQLAEVIYNSLYLGLDIPKGKHAIRFLCLEKEDFYYPLDRSDDYT
A4N5E7	MITVFGLKSKLSPRREQLAEVIYNSLHLGLDIPKGKHAIRFLCLEKEDFYYPFDRSDDYT

E2PB85	GGTGGAATGGTTATT**AGGAGA**AAAAA**ATG**ATTAGTGTATATGGATTAAAACAAACCTTAG
W0R8J4	AGTTCAATGGCTATT**GGAGG**CAAAAA**ATG**ATTAGTGTTTACGGATTAAAACACACACTTG
I3DIP6	TGGCTATTAGCTCGT**AAGGAGA**CATT**ATG**ATTACCGTTTATGGTTTAAAAAAATCACTTG
I3DR40	TTTAACCGCACTTT**AAAGGAGA**AAAA**ATG**ATCACCGTATTCGGACTTAAATCCAAACTCG
A4N5E7	TTTAACCGCACTTT**AAAGGAGA**AAAA**ATG**ATTACTGTATTCGGACTTAAATCCAAACTTT

A. **Top.** Multiple sequence alignment of five TSF homologs for which Met-1 is not followed by a proline, which is a deviation from the signature feature of TSF members. However, these sequences clearly align very well. **Bottom**. For all five sequences, their annotated start codons are associated with a Shine-Dalgarno-type sequence, indicating that the annotated start codon is likely the correct one. Indeed, no alternative start codons can be found nearby in which Met

would be followed by Pro and where this alternative start codons is also associated with a Shine-Dalgarno-type sequence.

An example of two misannotated sequences missing an N-terminal Pro are shown in B) and the same examples in which the misannoation has been corrected by identification of the true Met1 site is shown in C).

```
A0A0C1MDE4      -----MPIVNIDLIA-RSQDQLKALVQDVTTAVTKNTGAPAEHVHVILREMQPNRYGVA
H1LG62          MEEFIMPIVDIHLIA-RSQAQLKGLVEDVTAAVVKNTGAPAEHVHVILSEMQKDRYSVG
C0WTL0          -----MPIVNIQLIA-RSQDQLKALVADVTAAVVKDTGAPAEHVHVILDEMQKNRYSVG
J1GJS3          -----MPYVNIRVTREGVSAEQKLALIEGVTDLLEQVLNKKPADTFVVIDEVETDNWGV
F8G1U2          -----------MTNEGVSAEHKRQLIEQTTRMLEQVLGKPPASTFVVIEEVPTDNWGVG
A0A0P7D6Z0      -----MPYVHIRVTDEGVSAEHKRQLIEQTTCMLERVLGKPPASTFVVIEEVPTDNWGV
```

```
A0A0C1MDE4      TAAGAAGAGAAGGGAACGAAACTCAATGCCAATCGTAAACATCGACTTAAT
H1LG62          GGCGTATACTTTTTTAAACTAAAAAATGGAGGAATTTATTATGCCAATTGT
C0WTL0          TTAAAAAATTTGGAGGAACCTCATCATGCCAATCGTAAATATTCAACTTAT
J1GJS3          GGGGTTTGGTAACAGGAGAGCCGCTATGCCATACGTTAATATTCGCGTCAC
F8G1U2          AACCATGCCTTATGTCCATATTCGCGTGACCAATGAAGGCGTCAGTGCTGA
A0A0P7D6Z0      GGCATTAACAGAGGAGCGAGCAACCATGCCTTATGTCCATATTCGCGTGAC
```

B. Top. Multiple sequence alignment of six protein sequences from the TSF. For two of these sequences, Met-1 (in red) is not followed by Pro. However, their Met-1 is not aligned with Met-1 of the other four sequences, which are their closest homologs in the TSF. This is a clear sign of start codon misannotation. **Bottom.** The start of the DNA sequence of the genes of the six homologs, including the genomic DNA sequence directly upstream of the annotated start codon (in red). Underlined and in bold are the Shine-Dalgarno-type sequences that designate the presence of a ribosomal binding-site. Clearly, the annotated start codons of the two protein sequences for which Met-1 is not followed by Pro, are not associated with a Shine-Dalgarno-type sequence. However, an alternative potential start codon (in green) is seen nearby in the DNA sequence.

A0A0C1MDE4	MPIVNIDLIA-GRSQDQLKALVQDVTTAVTKNTGAPAEHVHVILREMQPNRYGVAGVLKS
H1LG62	MPIVDIHLIA-GRSQAQLKGLVEDVTAAVVKNTGAPAEHVHVILSEMQKDRYSVGGVLKS
C0WTL0	MPIVNIQLIA-GRSQDQLKALVADVTAAVVKDTGAPAEHVHVILDEMQKNRYSVGGVLKS
J1GJS3	MPYVNIRVTREGVSAEQKLALIEGVTDLLEQVLNKKPADTFVVIDEVETDNWGVNRESVS
F8G1U2	MPYVHIRVTNEGVSAEHKRQLIEQTTRMLEQVLGKPPASTFVVIEEVPTDNWGVGGETVT
A0A0P7D6Z0	MPYVHIRVTDEGVSAEHKRQLIEQTTCMLERVLGKPPASTFVVIEEVPTDNWGVGGETVT

A0A0C1MDE4	TAAGAA**GAGAAGGGAA**CGAAACTCA**ATG**CCAATCGTAAACATCGACTTAAT
H1LG62	AAACTAAAAAAT**GGAGGAA**TTTATT**ATG**CCAATTGTAGATATTCATTTAAT
C0WTL0	TTAAAAAATTT**GGAGGAA**CCTCATC**ATG**CCAATCGTAAATATTCAACTTAT
J1GJS3	GGGGTTTGGTAAC**AGGAGA**GCCGCT**ATG**CCATACGTTAATATTCGCGTCAC
F8G1U2	GGCATTAAC**AGAGGAGCGA**GCAACC**ATG**CCTTATGTCCATATTCGCGTGAC
A0A0P7D6Z0	GGCATTAAC**AGAGGAGCGA**GCAACC**ATG**CCTTATGTCCATATTCGCGTGAC

C. Top. Multiple sequence alignment of the six protein sequences of panel B, where the alternative start codon of the two non-Pro-1 sequences is used as the true start codon. Clearly, Met-1 is now followed by Pro. **Bottom**. The aligned new set of DNA sequences. The two alternative start codons are now associated with a Shine-Dalgarno-type sequence, indicating that the alternative start codons are likely the actual start codons of the genes. The fact that Met-1 is followed by a proline, is additional evidence that the start codons are now correctly annotated.

File S1. PDB codes of structures used in the structure similarity network provided in Figure 8.

1DPT
1FIM
1GYX
1HFO
1MFI
1MIF
1MWW
1OTF
1OTG
1U9D
1UIZ
2AAG
2OP8
2ORM
2OS5
2WKB
2WKF
2X4K
2XCZ
3ABF
3B64
3C6V
3E6Q
3EJ3, chain B (*cis*-CaaD)
3EJ3, chain C (*cis*-CaaD)
3FWT
3GAD
3KER
3M20
3MB2, chain B (*cis*-CaaD)
3MB2, chain C (*cis*-CaaD)
3MF8
3N4G (CgX)
3RY0
3T5S
4DH4
4FAZ
4FDX
4JCU
4JJ9
4LHP
4LKB
4M1A
4OTA (4-OT)
4U5P
5UIF (Linker 1)
5UNQ (Linker 2)
6BLM (Fused 4-OT)

Notes: Structures used in **Figure 8** and in the MSA for **Figure 5** differ for 4-OT and *cis*-CaaD. For 4-OT, PDB 1BJP is used in the MSA for **Figure 5** and PDB 4OTA is used in **Figure 8**. For *cis*-CaaD, PDB 2FLZ is used in the MSA for **Figure 5** and PDBs 3EJ3 and 3MB2 are used in **Figure 8**. Two chains each from *cis*-CaaD structures 3EJ3 and 3MB2 were used in **Figure 8** as these proteins are organized as part of physiological heterohexamers in which the B and C chains are different.
